# Postpartum breast cancer: evidence for a distinct phenotype

**DOI:** 10.1093/jnci/djag003

**Published:** 2026-01-09

**Authors:** Mark E Sherman, Lola Etievant, Robert A Vierkant, Stacey J Winham, Kathryn J Ruddy, Laura Pacheco-Spann, Daniel P Wickland, Nicole Cruz-Reyes, Melody Stallings-Mann, Derek Radisky, E Aubrey Thompson, Jennifer M Kachergus, Ji Shi, Shoshana M Rosenberg, Craig Snow, Gregory J Kirkner, Lidia Schapira, Jeffrey M Peppercorn, Steven Come, Virginia F Borges, Ellen Warner, Laura C Collins, Ann H Partridge, Ruth M Pfeiffer

**Affiliations:** Quantitative Health Sciences, Mayo Clinic, Jacksonville, FL, United States; Division of Cancer Epidemiology and Genetics, National Cancer Institute, National Institutes of Health, Bethesda, MD, United States; Quantitative Health Sciences, Mayo Clinic, Rochester, MN, United States; Quantitative Health Sciences, Mayo Clinic, Rochester, MN, United States; Department of Oncology, Mayo Clinic, Rochester, MN, United States; Quantitative Health Sciences, Mayo Clinic, Jacksonville, FL, United States; Quantitative Health Sciences, Mayo Clinic, Jacksonville, FL, United States; Department of Cancer Biology, Mayo Clinic, Jacksonville, FL, United States; Department of Cancer Biology, Mayo Clinic, Jacksonville, FL, United States; Department of Cancer Biology, Mayo Clinic, Jacksonville, FL, United States; Department of Cancer Biology, Mayo Clinic, Jacksonville, FL, United States; Department of Cancer Biology, Mayo Clinic, Jacksonville, FL, United States; Department of Cancer Biology, Mayo Clinic, Jacksonville, FL, United States; Medical Oncology, Dana-Farber Cancer Institute, Boston, MA, United States; Breast Oncology Program, Dana-Farber Brigham Cancer Center, Boston, MA, United States; Medical Oncology, Dana-Farber Cancer Institute, Boston, MA, United States; Breast Oncology Program, Dana-Farber Brigham Cancer Center, Boston, MA, United States; Medical Oncology, Dana-Farber Cancer Institute, Boston, MA, United States; Breast Oncology Program, Dana-Farber Brigham Cancer Center, Boston, MA, United States; Department of Medicine, Division of Medical Oncology, Stanford University, Stanford, CA, United States; Department of Medicine, Division of Medical Oncology, Massachusetts General Hospital, Boston, MA, United States; Medical Oncology, Beth Israel Deaconess Medical Center, Boston, MA, United States; Medical Oncology, University of Colorado Anschutz Medical Campus, Aurora, CO, United States; Department of Medicine, Division of Medical Oncology, Odette Cancer Centre, Sunnybrook Health Sciences Centre, Toronto, ON, Canada; Pathology, Beth Israel Deaconess Medical Center, Boston, MA, United States; Medical Oncology, Dana-Farber Cancer Institute, Boston, MA, United States; Breast Oncology Program, Dana-Farber Brigham Cancer Center, Boston, MA, United States; Division of Cancer Epidemiology and Genetics, National Cancer Institute, National Institutes of Health, Bethesda, MD, United States

## Abstract

**Background:**

The incidence of early onset breast cancers (BCs) has increased, paralleling rising trends in delayed childbearing. We hypothesize that a distinct postpartum BC (PPBC) subtype, identifiable by time since last birth (TSLB) and biomarker expression, contributes to this trend.

**Methods:**

We applied GeoMx Digital Spatial Profiling (DSP) to measure associations between TSLB and 71 proteins in 640 BCs from women aged ≤40 years included in the Young Women’s Breast Cancer Study. We analyzed data using univariable linear regression and multivariable sliced inverse regression to account for higher order interactions among biomarkers.

**Results:**

In keratin-rich segments, progesterone receptor (PR) (*P* = 1.00 × 10^−4^) and PTEN (*P* = 1.00 × 10^−3^) were associated with longer TSLB; multivariable analyses revealed positive associations for GZMB (*P* = 4.00 × 10^−4^), SMA (*P* = 1.00 × 10^−4^), and NF-1 (*P* = 1.00 × 10^−3^). In keratin-poor segments, univariable significant positive associations were found for PR (*P* = 2.00 × 10^−4^) and PTEN (*P* = 1.00 × 10^−3^), whereas CD20 (*P* = 3.00 × 10^−4^) and CTLA4 (*P* = 4.00 × 10^−5^) were negatively associated; multivariable significant associations were found for fibronectin (*P* = 3.00 × 10^−5^) and pan-Akt (*P* = 1.00 × 10^−3^). Associations persisted after adjustment for multiple comparisons and BC molecular subtypes. Associations including for PR, PTEN, and CD20 were strongest among women with the shortest TSLB. OPAL multiplex immunofluorescence assays for PR, PTEN, CD20, SMA, and CTLA4 replicated several DSP findings, particularly when stratified by subtype and with compartment matching. In TCGA, RNA species linked to proteins associated with TSLB correlated strongly with a T-cell exhaustion signature previously linked to poor prognosis among premenopausal women.

**Conclusion:**

These data support PPBC as a biologically coherent phenotype defined by TSLB and biomarker profile, with potential implications for prevention and therapy.

## Introduction

Breast cancer (BC) exhibits a bimodal age-specific incidence pattern, peaking at 50 and 70 years,^1^ and persisting in strata defined by estrogen receptor (ER), progesterone receptor (PR), and human epidermal growth factor receptor 2 (HER2) expression.^1^^,^^2^ Moreover, the clinical phenotypes of early onset and late onset BCs with similar biomarker expression differ. Specifically, ER-positive/HER2-negative BCs in young women demonstrate more aggressive pathologic features and worse outcomes than those in older women, even after adjusting for grade and stage.^3^^,^^4^

Incidence rates of early onset BCs rise after childbirth, peak 5 years later, and then return slowly to baseline for ER-positive BCs, while remaining elevated for ER-negative BCs.^5^ Among women with a family history of BC or late age at first birth, 2 established BC risk factors, these rates are even higher.^5^ In the United States, increasing incidence rates of premenopausal ER-positive BCs parallel trends in delayed childbearing,^6^^,^^7^ suggesting a potential relationship and highlighting an unmet need for prevention.

In rodent models, postpartum involution at weaning, which restores the mammary gland to a nongravid state, is associated with a procarcinogenic, immune suppressed wound healing response, which can be inhibited with anti-inflammatory agents.^8^^,^^9^ Among women, an analogous process transforms the hyperplastic pregnant breast to a nongravid state and may contribute to increased BC risk after childbirth.^8^^,^^9^ Compared with normal breast tissues of nulliparous women, tissues of parous women demonstrate a greater content of lobules, a transient increase in immune cells, and a different pattern of RNA expression, which evolves over years,^10-14^ potentially conferring short-term increases in BC risk, followed by delayed protection. Mechanisms proposed to account for poor outcomes among postpartum BC (PPBC) patients include lymphangitic spread,^15^^,^^16^ suppressed immune responses,^17^ stromal remodeling and a propensity for hepatic metastases.^8^ Thus, disentangling effects of pregnancy, lactation and postpartum involution and remodeling is important because these events alter breast parenchyma, immunity, and BC risk.

Immune suppression may be important in the genesis of PPBCs. Preclinical data show that progesterone-mediated upregulation of immune checkpoint B7-H4 is linked to immune tolerance of the fetus during pregnancy and to immune evasion in BC.^18^ B7-H4 is expressed in 90% of triple-negative BCs (TNBCs) and agents targeting this factor have shown promise in early clinical trials.^19^ Treatment of postmenopausal BCs with a high ratio of PRA to PRB with the PR antagonist mifepristone decreased B7-H4 transcripts, increased immune cell infiltration, and reduced proliferation.^20^ Thus, there is a potential link between PR-mediated gene expression and immune suppression in human physiology and BC. Moreover, Egelston et al^21^ defined a T-cell exhaustion RNA signature that predicts a poor prognosis for premenopausal (but not postmenopausal) ER-positive BCs; however, this report did not evaluate time since last birth (TSLB).

A bioinformatics analysis of RNA sequencing of 16 ER-positive BCs from women aged ≤45 years found that BCs in parous women showed increased expression of genes related to cell cycling, T cell presence-activation and suppression, cell death, and DNA repair, and reduced *ESR1* and *TP53* signaling.^22^ These signatures overlapped with those of normal pregnancy and postpartum involution, suggestively linking carcinogenesis and reproductive events. A gene signature derived from these data based on upregulation of immune exhaustion and *E2F1* regulons and downregulation of *ESR1* and *p53* pathways predicted significantly higher overall mortality among 311 BC patients aged ≤45 years reported in the literature; however, TSLB was not addressed in these analyses. Similarly, an analysis of a multiracial BC cohort of women aged ≤50 years found that BCs diagnosed within 10 years of childbirth contained more B cell, T cell, and NK marker expression than BCs of nulliparas.^23^ In aggregate, data suggest that immune responses may be critical in PPBC development.^19^^,^^22^^,^^24^^,^^25^

It is proposed that PPBCs may include BCs diagnosed 5-10 years postpartum^26^ and should be distinguished from BCs diagnosed during pregnancy, which may have a better prognosis.^26^^,^^27^ While childbirth is linked to a transient increase in BC risk and potentially to poor prognosis,^15^^,^^28-30^ criteria for defining a unified category of PPBC are lacking, limiting research to improve prevention and therapy. Here, we hypothesize that a subset of early onset BCs represents a distinct PPBC subtype, definable by integrating TSLB with molecular markers. We reason that identification of markers associated with TSLB, after adjustment for confounders, could support efforts to define PPBC as a distinct clinical entity.

## Methods

### Study population

The Young Women’s Breast Cancer Study is a multi-institutional cohort started in 2006 that enrolled women with incident BC diagnosed at aged ≤40 years.^31^^,^^32^ Women were recruited by mail, consented to provide access to medical records, completed a questionnaire, and donated blood and tissue. Tissue microarrays (TMAs) of BCs obtained before treatment were available for 336 parous women with recorded TSLB (*n* = 789 cores) and 268 nulliparas (*n* = 635 cores). The main study received institutional review board approval from Dana-Farber/Harvard Cancer Center and other participating centers, including Mayo Clinic.

Surrogate BC molecular subtypes were defined using pathology reports as luminal A if ER-positive and/or PR-positive, HER2- negative and grade 1 or 2; luminal B if hormone receptor positive and HER2-positive or HER2-negative and grade 3; HER2-enriched if hormone receptor negative and HER2-positive (immunohistochemistry 3+ or fluorescent in situ hybridization amplified); and triple-negative (TNBC) if all assays were negative.

### Marker measurements

We used NanoString GeoMx Digital Spatial Profiling (DSP) to apply 4 immunofluorescence markers (keratin, SYTO13, CD68, and CD45) for visualization and selection of circular regions of interest (ROIs) measuring 600 µm in diameter, analyzed as keratin-rich (ie, epithelial-rich) and keratin-poor (ie, epithelial-poor) segments. Region of interest placement covered most cells within each core.^33^ A cocktail of 71 bar-coded antibodies was applied, followed by laser illumination of ROIs to release tags from antibodies bound to targets and measured on the *n*-counter. Up to 8 ROIs were available per woman (see Supplementary Methods).

### Biomarker replication (multiplex immunofluorescence [OPAL])

To replicate key DSP associations with TSLB, we performed OPAL multiplex immunofluorescence for PR, CD20, PTEN, CTLA4, and SMA on a single TMA section. After excluding 224 cores because of artifacts, fewer than 100 cytokeratin-positive cells for epithelial markers or total cellularity less than 2 SDs below the mean cell count for nonepithelial markers, 1237 cores were evaluable. For interpretability across platforms, OPAL cytokeratin (CK)^+^-gated epithelium was treated as the counterpart to DSP keratin-rich segments, and OPAL stromal/CK^−^ as the counterpart to DSP keratin-poor segments. OPAL staining (Akoya Biosciences) was performed on 4 µm sections using iterative antigen retrieval and tyramide signal amplification (see Supplementary Methods).

### Statistical analysis

The main analysis tested associations of TSLB and biomarker expression among 336 parous women with recorded TSLB, evaluated continuously and as an ordinal variable, and among the entire study group, including nulliparas assigned the longest TSLB. Positive slopes indicate higher marker expression with longer TSLB.

Demographic and clinical characteristics of the parous participants were compared across BC subtypes using χ^2^ and Kruskal-Wallis tests. We assessed associations of reproductive factors with biomarker expression using linear mixed models, adjusted for age at diagnosis and accounting for multiple ROIs within a woman by fitting a per-patient random intercept term using an exchangeable variance-covariance correlation structure.

To identify potential confounders of the relationship between TSLB and each biomarker level, we fit linear regression models to the continuous TSLB variable with each potential confounder separately. Characteristics for which univariable significant associations with TSLB were found were then jointly included in a linear regression model (continuous TSLB) and a polytomous regression model (ordinal TSLB), and we used stepwise selection to identify the minimal set of confounders. Finally, we fit linear regression models adjusted for the identified confounders to each of the 53 log-transformed biomarkers in keratin-rich segments and computed residuals from these models. We repeated the analysis for 48 log-transformed biomarkers in keratin-poor segments. Correlations stemming from multiple ROIs from the same woman were accommodated in the variance calculation by using a robust sandwich variance estimate (R packages *sandwich* and *vcovCL*). Student *t*-tests were used to assess the significance of the associations.

To accommodate correlations among markers, and assess multivariate associations, we used Sliced Inverse Regression (SIR)^34^ to obtain linear combinations of the biomarker residuals associated with the categories of TSLB as the outcome. A marginal dimension test^35^ was used to decide how many SIR linear components were associated with TSLB, wherein an estimated dimension larger than 1 indicates quadratic or higher order relationships among markers. Importance of each marker in the SIR linear components was assessed with the coordinate hypothesis test,^36^ thereby identifying markers associated with TSLB, while accommodating complex interactions and correlations among the markers. We used a Bonferroni adjustment to accommodate multiple testing.

Replication was performed by OPAL per above using linear regression with the log-transformed markers as the outcome and categorical TSLB fitted with a trend for overall BC and stratified by molecular subtype. Models were adjusted for ovarian cancer or BC family history, age at BC diagnosis and menopausal status to assess marker levels by TSLB.

In additional analyses, we assessed whether adjusting for molecular subtypes affected conclusions for associations of TSLB and biomarkers. As a quality check, we affirmed separability of molecular subtypes by computing pairwise Pearson correlation coefficients of the biomarker residuals in keratin-rich segments and used Uniform Manifold Approximation and Projection^37^ to investigate clustering by BC subtype. We used linear regression models with each log-transformed biomarker as the outcome adjusted for the confounders to evaluate trends in biomarker levels by categories of TSLB, including nulliparas as the category with the longest TSLB. We assessed whether transcripts of all genes corresponding to proteins associated with TSLB were associated with a T-cell exhaustion signature in BCs (*n* = 79) and in normal-tissue samples (*n* = 12) among women aged ≤40 years in The Cancer Genome Atlas (TCGA).^21^ The Cancer Genome Atlas expression data (counts) were downloaded from the Genomic Data Commons data portal of the National Cancer Institute (https://portal.gdc.cancer.gov). Differential expression analysis was performed using R package DESeq2 (version 1.42.1) to identify associations between gene expression and age at diagnosis.^38^ T-cell exhaustion (T_Ex_) scores were estimated with weighted gene expression levels as reported^21^ using the sig.score function of the R package genefu (version 2.34.0) with default settings. Pearson correlation between T_Ex_ score and TSLB biomarker expression in TCGA were calculated using the cor function in R, with the Benjamini-Hochberg method applied for multiple-testing correction.^39^ The R package ComplexHeatmap (version 2.18.0) was used to visualize the correlations.

## Results

### Participants

The mean age at diagnosis (SD) of participants was 36.6 (3.1) years, and the mean TSLB (SD) was 4.4 (4.0) years. Of 336 BCs among parous women, 104 were luminal A; 148 luminal B; 37 TNBC, and 26 HER2-enriched with 21 missing subtype (Table 1). Compared with other subtypes, patients with TNBC were more likely to have reported an earlier age at menarche (*P* = .01), not to have breastfed (*P* = .01), and to have a first degree relative with breast and/or ovarian cancer (*P* = .04).

**Table 1.** Demographic and clinical characteristics of 336 parous study participants with known time since last birth^a^ by breast cancer subtypes.

	Overall^b^	Missing	Luminal A	Luminal B	HER2	TNBC	*P* ^c^
	(*n* = 336)	(*n* = 21)	(*n* = 104)	(*n* = 148)	(*n* = 26)	(*n* = 37)	
Age at diagnosis							
Mean (SD)	36.6 (3.06)	37.5 (2.29)	36.8 (2.86)	36.2 (3.32)	36.8 (2.63)	36.6 (3.08)	0.54
Age at menarche, years							
<12	45 (13.4%)	3 (14.3%)	10 (9.6%)	15 (10.1%)	5 (19.2%)	12 (32.4%)	0.01
12-13	127 (37.8%)	14 (66.7%)	43 (41.3%)	53 (35.8%)	7 (26.9%)	10 (27.0%)	
14+	55 (16.4%)	3 (14.3%)	16 (15.4%)	27 (18.2%)	7 (26.9%)	2 (5.4%)	
Missing	109 (32.4%)	1 (4.8%)	35 (33.7%)	53 (35.8%)	7 (26.9%)	13 (35.1%)	
Number of pregnancies prediagnosis							
0	1 (0.3%)	0 (0%)	0 (0%)	1 (0.7%)	0 (0%)	0 (0%)	0.55
1	53 (15.8%)	3 (14.3%)	14 (13.5%)	25 (16.9%)	2 (7.7%)	9 (24.3%)	
2	128 (38.1%)	8 (38.1%)	47 (45.2%)	51 (34.5%)	11 (42.3%)	11 (29.7%)	
3-5	141 (42.0%)	10 (47.6%)	40 (38.5%)	63 (42.6%)	11 (42.3%)	17 (45.9%)	
Missing	13 (3.9%)	0 (0%)	3 (2.9%)	8 (5.4%)	2 (7.7%)	0 (0%)	
Number of live children prediagnosis							
0	1 (0.3%)	0 (0%)	1 (1.0%)	0 (0%)	0 (0%)	0 (0%)	0.6
1	92 (27.4%)	5 (23.8%)	26 (25.0%)	43 (29.1%)	4 (15.4%)	14 (37.8%)	
2	161 (47.9%)	10 (47.6%)	51 (49.0%)	70 (47.3%)	14 (53.8%)	16 (43.2%)	
3+	68 (20.2%)	5 (23.8%)	23 (22.1%)	26 (17.6%)	7 (26.9%)	7 (18.9%)	
Missing	14 (4.2%)	1 (4.8%)	3 (2.9%)	9 (6.1%)	1 (3.8%)	0 (0%)	
Number of miscarriages prediagnosis							
0	227 (67.6%)	14 (66.7%)	76 (73.1%)	98 (66.2%)	16 (61.5%)	23 (62.2%)	0.05
1	68 (20.2%)	5 (23.8%)	13 (12.5%)	36 (24.3%)	5 (19.2%)	9 (24.3%)	
2+	26 (7.7%)	1 (4.8%)	12 (11.5%)	5 (3.4%)	3 (11.5%)	5 (13.5%)	
Missing	15 (4.5%)	1 (4.8%)	3 (2.9%)	9 (6.1%)	2 (7.7%)	0 (0%)	
Number of stillbirths prediagnosis							
0	317 (94.3%)	20 (95.2%)	100 (96.2%)	136 (91.9%)	25 (96.2%)	36 (97.3%)	0.6
1	5 (1.5%)	0 (0%)	1 (1.0%)	3 (2.0%)	0 (0%)	1 (2.7%)	
Missing	14 (4.2%)	1 (4.8%)	3 (2.9%)	9 (6.1%)	1 (3.8%)	0 (0%)	
Number of abortions prediagnosis							
0	263 (78.3%)	17 (81.0%)	83 (79.8%)	112 (75.7%)	22 (84.6%)	29 (78.4%)	0.61
1+	57 (17.0%)	3 (14.3%)	17 (16.3%)	26 (17.6%)	3 (11.5%)	8 (21.6%)	
Missing	16 (4.8%)	1 (4.8%)	4 (3.8%)	10 (6.8%)	1 (3.8%)	0 (0%)	
Breastfeeding status							
Breastfed	228 (67.9%)	15 (71.4%)	76 (73.1%)	95 (64.2%)	23 (88.5%)	19 (51.4%)	0.01
Did not breastfeed	96 (28.6%)	6 (28.6%)	25 (24.0%)	45 (30.4%)	2 (7.7%)	18 (48.6%)	
Missing	12 (3.6%)	0 (0%)	3 (2.9%)	8 (5.4%)	1 (3.8%)	0 (0%)	
Time since last birth (continuous)							
Mean (SD)	4.44 (3.96)	5.33 (3.94)	4.96 (4.32)	4.33 (3.93)	3.00 (2.56)	3.89 (3.63)	0.11
TSLB (categorical)							
0-3 years	135 (40.2%)	8 (38.1%)	36 (34.6%)	57 (38.5%)	13 (50.0%)	21 (56.8%)	0.34
3-5 years	97 (28.9%)	4 (19.0%)	33 (31.7%)	46 (31.1%)	9 (34.6%)	5 (13.5%)	
6-10 years	80 (23.8%)	8 (38.1%)	27 (26.0%)	34 (23.0%)	3 (11.5%)	8 (21.6%)	
11-21 years	24 (7.1%)	1 (4.8%)	8 (7.7%)	11 (7.4%)	1 (3.8%)	3 (8.1%)	
Menopause status							
No	189 (56.3%)	20 (95.2%)	66 (63.5%)	73 (49.3%)	16 (61.5%)	14 (37.8%)	0.11
Not sure	107 (31.8%)	0 (0%)	29 (27.9%)	52 (35.1%)	8 (30.8%)	18 (48.6%)	
Yes	26 (7.7%)	1 (4.8%)	5 (4.8%)	14 (9.5%)	1 (3.8%)	5 (13.5%)	
Missing	14 (4.2%)	0 (0%)	4 (3.8%)	9 (6.1%)	1 (3.8%)	0 (0%)	
Family history of ovarian and/or BC							
No	282 (83.9%)	16 (76.2%)	91 (87.5%)	129 (87.2%)	20 (76.9%)	26 (70.3%)	0.04
Yes	54 (16.1%)	5 (23.8%)	13 (12.5%)	19 (12.8%)	6 (23.1%)	11 (29.7%)	
Smoking status							
Never	207 (61.6%)	12 (57.1%)	70 (67.3%)	87 (58.8%)	16 (61.5%)	22 (59.5%)	0.67
Former	108 (32.1%)	8 (38.1%)	27 (26.0%)	51 (34.5%)	8 (30.8%)	14 (37.8%)	
Current	6 (1.8%)	0 (0%)	3 (2.9%)	2 (1.4%)	0 (0%)	1 (2.7%)	
Missing	15 (4.5%)	1 (4.8%)	4 (3.8%)	8 (5.4%)	2 (7.7%)	0 (0%)	
Alcohol consumption status							
Never or former	94 (28.0%)	4 (19.0%)	26 (25.0%)	44 (29.7%)	3 (11.5%)	17 (45.9%)	0.06
Current	226 (67.3%)	16 (76.2%)	74 (71.2%)	95 (64.2%)	21 (80.8%)	20 (54.1%)	
Missing	16 (4.8%)	1 (4.8%)	4 (3.8%)	9 (6.1%)	2 (7.7%)	0 (0%)	
BMI at diagnosis							
16.6-20.9	70 (20.8%)	7 (33.3%)	25 (24.0%)	21 (14.2%)	5 (19.2%)	12 (32.4%)	0.28
21-24.9	155 (46.1%)	8 (38.1%)	50 (48.1%)	72 (48.6%)	11 (42.3%)	14 (37.8%)	
25-47.5	99 (29.5%)	6 (28.6%)	26 (25.0%)	47 (31.8%)	9 (34.6%)	11 (29.7%)	
Missing	12 (3.6%)	0 (0%)	3 (2.9%)	8 (5.4%)	1 (3.8%)	0 (0%)	
Race							
White	303 (90.2%)	20 (95.2%)	94 (90.4%)	133 (89.9%)	22 (84.6%)	34 (91.9%)	0.25
Non-White	28 (8.3%)	1 (4.8%)	10 (9.6%)	10 (6.8%)	4 (15.4%)	3 (8.1%)	
Missing	5 (1.5%)	0 (0%)	0 (0%)	5 (3.4%)	0 (0%)	0 (0%)	
Ethnicity (Hispanic)							
No	312 (92.9%)	19 (90.5%)	101 (97.1%)	132 (89.2%)	24 (92.3%)	36 (97.3%)	0.24
Yes	19 (5.7%)	2 (9.5%)	2 (1.9%)	12 (8.1%)	2 (7.7%)	1 (2.7%)	
Missing	5 (1.5%)	0 (0%)	1 (1.0%)	4 (2.7%)	0 (0%)	0 (0%)	
Number of repeated regions of interest (ROIs)							
1	63 (18.8%)	8 (38.1%)	24 (23.1%)	24 (16.2%)	5 (19.2%)	2 (5.4%)	0.1
2	119 (35.4%)	9 (42.9%)	37 (35.6%)	53 (35.8%)	8 (30.8%)	12 (32.4%)	
3	143 (42.6%)	4 (19.0%)	42 (40.4%)	62 (41.9%)	12 (46.2%)	23 (62.2%)	
4+	11 (3.3%)	0 (0%)	1 (1.0%)	9 (6.1%)	1 (3.8%)	0 (0%)	

Number of pregnancies more than 5 years before BC diagnosis was not utilized because 41% of the women with small  TSLB had missing values.

Abbreviations: BC = breast cancer; BMI = body mass index; ROI = region of interest; SD = standard deviation; TSLB = time since last birth.

a Thirty-six parous study participants with missing TSLB were removed from the analytic dataset, leaving 336 women for analysis.

b Parous study participants with known TSLB.

c *P*-values from χ^2^ or Kruskal-Wallis tests for comparison across BC subtypes.

### Association of BC risk factors with biomarkers among parous women

Age at diagnosis, number of pregnancies prediagnosis, number of live children prediagnosis, breastfeeding history, menopausal status, and family history of ovarian cancer or BC were marginally associated with TSLB and identified as potentially confounding the biomarker associations with TSLB (Table S1 and Figure S1). Body mass index at enrollment was included due to its known BC risk association.

### Biomarkers associated with TSLB: keratin-rich segments

Nominal associations with TSLB (*P*≤.05) were found for 19 biomarkers (Table S2), with PR (*P* = 1.00 × 10^−4^) and PTEN (*P* = 1.00 × 10^−3^) remaining significantly positively associated after adjustment for multiple testing, and CD20 borderline significantly negatively related (Table 2: markers *P* ≤ .01). Using SIR to analyze all markers jointly, 3 components were identified, indicating complex interactions among biomarkers. SIR analysis revealed 17 markers marginally associated with TSLB of which GZMB (*P* = 4.00 × 10^−4^) and SMA (*P* = 1.00 × 10^−4^) showed significant positive associations and NF-1(*P* = 1.00 × 10^−3^) showed a significant negative association after adjusting for multiple comparisons.

### Biomarkers associated with TSLB: keratin-poor segments

We identified 18 nominal associations of TSLB and single biomarkers in keratin-poor segments (Table S3: all markers), of which 4 remained significant after adjustment for multiple comparisons; specifically, positive associations were found for PR (*P* = 2.00 × 10^−4^) and PTEN (*P* = 1.00 × 10^−3^) and negative associations for CD20 (*P* = 3.00 × 10^−4^) and CTLA4 (*P* = 4.00 × 10^−5^) (Table 3: markers *P* ≤ .01). In SIR analyses, 12 markers were nominally associated with TSLB; fibronectin (*P* = 3.00 × 10^−5^) and pan-Akt (*P* = 1.00 × 10^−3^) remained significant after multiple testing adjustment.

**Table 3.** Individual associations between time since last birth (TSLB) and the 48 biomarkers,^a^ and multivariate associations between time since last birth (TSLB) and the 48 biomarker residuals,^b^ in the 335 parous study participants with known TSLB,^c^ in keratin-poor segments.

Individual associations			Multivariate associations
Biomarker	Slope estimate from marginal linear regression^d^	*P*-value from test of significance in marginal linear regression^d^	*P*-value from test of significance^e^ in the 2 SIR^f^ components
Bcl2	0.04	3.00E-03	.28
BCL6	0.03	5.00E-03	.84
CD20	−0.04	**3.00E-04**	.04
CD44	−4.00E-03	.78	2.00E-03
Cleaved Caspase 9	0.02	2.00E-03	.63
CTLA4	−0.07	**4.00E-5**	.07
Fibronectin	0.05	.01	**3.00E-05**
NF1	0.05	.01	.03
Pan-AKT	0.02	.03	**1.00E-03**
PARP	0.03	5.00E-03	.48
PD_1	0.02	4.00E-03	.93
Phospho-AKT1-S473	0.02	.01	.99
PR	0.06	**2.00E-04**	.12
PTEN	0.04	**1.00E-03**	.01
Tim_3	0.03	.01	.06

Restricted to biomarkers with *P* ≤ .01 (see Supplementary Material for all markers).

Bold reflects significant at the Bonferroni corrected level (0.05/48).

Abbreviations: BC = breast cancer; BMI = body mass index; ROI = region of interest; SIR = Sliced Inverse Regression; TSLB = time since last birth.

a Expression values of the biomarkers were log2-transformed checked for adequate quality. In the stromal dataset, 5 markers were additionally excluded, leaving 48 markers for analysis.

b Residuals from linear regressions of the 48 log-transformed biomarkers individually explained by age at diagnosis, number of live children prediagnosis, breastfeeding, menopause status, family history of ovarian or BC, and BMI in the parous study participants with known TSLB.

c Thirty-six parous study participants with missing TSLB removed from the analytic dataset.

d Continuous TSLB used in linear regression models with each individual biomarker as outcome and adjusting for age at diagnosis, number of live children prediagnosis, breastfeeding, menopause status, family history of ovarian or BC, and BMI in the parous study participants with known TSLB. Student *t*-tests were used to assess the significance of the associations.

e Wald-type tests testing the importance of each individual biomarker residual in all 3 SIR components obtained from the parous study participants with known TSLB.

f SIR components defined as weighted sums of the 48 biomarker residuals. Marginal dimension test indicated 2 SIR components were needed. Repeated ROIs of the 335 parous study participants with known TSLB considered as independent observations (*n* = 790).

### Biomarker replication analyses

**Figure 1.**
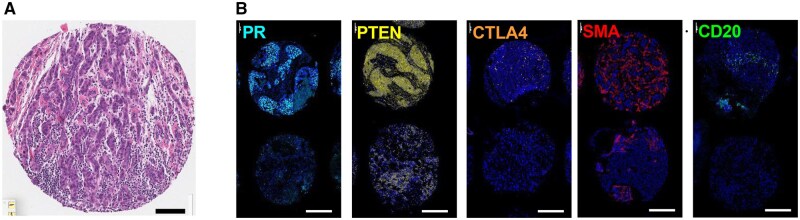
OPAL multiplex immunofluorescent staining of markers associated with time since last birth (TSLB). **(A)** Hematoxylin and eosin-stained core from the Young Women’s Breast Cancer Study tissue microarray (TMA). Scale bar, 100 μm. **(B)** OPAL multiplex immunofluorescent images showing PR, PTEN, CTLA4, SMA, and CD20 channels after spectral unmixing. Representative examples of relatively high (top) and low (bottom) expression are shown for each biomarker. Scale bar, 250 μm. OPAL staining was performed on a single 4-μm TMA section from 640 women; after quality control, 1,237 cores from parous and nulliparous participants remained evaluable. Marker-positive cells were identified using automated segmentation and phenotype thresholds derived from single-plex controls, and marker expressing cells were evaluated within CK-gated epithelial or stromal masks. Associations between TSLB and marker expression were estimated using regression models with TSLB as a predictor (parous-only and all-women models), adjusted for confounders and stratified by molecular subtype. Quantification of biomarker expression by TSLB among parous women and among nulliparas by BC molecular subtype, displayed as boxplots of log-transformed marker positive cells adjusted for total cell number in CK^+^ epithelium (PR, PTEN) and CK^−^/stromal compartments (CTLA4, SMA, CD20) are shown in Figure S2.

To support associations of top DSP markers with TSLB, we performed multiplex stains for CD20, CTLA4, PR, PTEN, and SMA on additional TMA sections (Figure 1). In parous-only models, TSLB was suggestively associated with CTLA4 (*P* = .067). When all women were analyzed (nulliparas included as the longest TSLB category), significant associations were found with TSLB for PR (*P* = .024) and PTEN in CK-positive cells (*P* = .027; Table 4). Because associations for markers varied by molecular subtype, and the admixture of subtypes varied by TSLB, we stratified analyses by subtype. These analyses revealed multiple statistically significant or suggestive associations of markers with TSLB among parous women; for luminal B, CTLA4 (*P* = .07) and CD20 (*P* = .10); for HER2-enriched, PR (*P* < .001) and PTEN (*P* = .09); and for TNBC, PR (*P* = .01) and SMA (*P* = .02). For all women (including nulliparas), luminal A was associated with PR (*P* = .03) and PTEN (*P* < .01); HER2-enriched with PTEN (*P* = .01) and SMA (*P* < .01) and TNBC with CD20 (*P* < .001), PTEN (*P* < .01) and SMA (*P* = .05) (Table S4, Figure S2). As an exploratory architectural readout, luminal A BCs exhibited elevated SMA^+^ cancer-associated fibroblasts (CAFs) within 25 µm of cytokeratin^+^ epithelium in women 5-10 years (*P* = .0139) and >10 years postpartum (*P* = .0132) vs nulliparous, consistent with *G*(*r*) proximity curves (Figure S3).

**Table 4.** OPAL multiplex immunofluorescence: associations between time since last birth (TSLB) and biomarker expression.

	Parous women only		All women (nulliparous treated as longest TSLB)	
Biomarker	Estimate (95% CI)	χ^2^	Estimate (95% CI)	χ^2^
CTLA4	0.058 (−0.004 to 0.000)	0.067	0.006 (−0.023 to 0.035)	0.691
PR	0.151 (−0.115 to 0.417)	0.266	0.139 (0.018 to 0.26)	0.024
SMA	0.037 (−0.072 to 0.147)	0.506	0.016 (−0.037 to 0.070)	0.547
CD20	0.139 (−0.093 to 0.372)	0.240	−0.070 (−0.166 to 0.027)	0.156
PTEN	0.110 (−0.165 to 0.386)	0.432	0.148 (0.017 to 0.279)	0.027

Linear models of log-transformed marker densities fitted with TSLB in ordered categories and a linear trend. Results are shown for parous women only and for all women (including nulliparous, treated as the longest TSLB). Positive estimates indicate higher expression with longer TSLB.

Abbreviations: CI = confidence interval; CK = cytokeratin; CAF = cancer-associated fibroblast.

### Distribution of breast cancer biomarkers

Uniform Manifold Approximation and Projection cluster analysis of the 53 biomarker residuals derived from analysis of keratin-rich segments demonstrated distinct clusters for luminal A, HER2-enriched and TNBC as expected. Luminal B subtypes were poorly discriminated, in part because HER2 expression contributed to this category (Figure S4). Pairwise-Pearson correlations of the residuals of the 53 log-transformed biomarker expression in keratin-rich segments among parous patients with known TSLB ranged from −0.36 to 0.87 (Figure S5).

### Additional analyses

In models additionally adjusted for BC molecular subtype, univariable *P*-values for CD20 remained significant and IDO-1, PTEN, and STING approached significance, and in multivariate analyses, fibronectin, GZMB, and SMA remained significant after adjusting for multiple comparisons. Characteristics of nulliparous women are presented in Table S5. In analyses of biomarker trends for ordinal categories of TSLB, 18 biomarkers were nominally significant, and CD20 (*P* = 1.00 × 10^−3^), CTLA4 (*P* = 1.00 × 10^−3^), PD-1 (*P* = 1.00 × 10^−3^), PR (*P* = 1.00 × 10^−4^), and PTEN (*P* = 5.00 × 10^−4^) remained significant after Bonferroni correction (Table S6). SIR analysis, including both parous and nulliparous women, identified 14 nominal biomarker associations and significant associations adjusted for multiple comparisons for SMA and an association approaching significance for GZMB.

**Figure 2.**
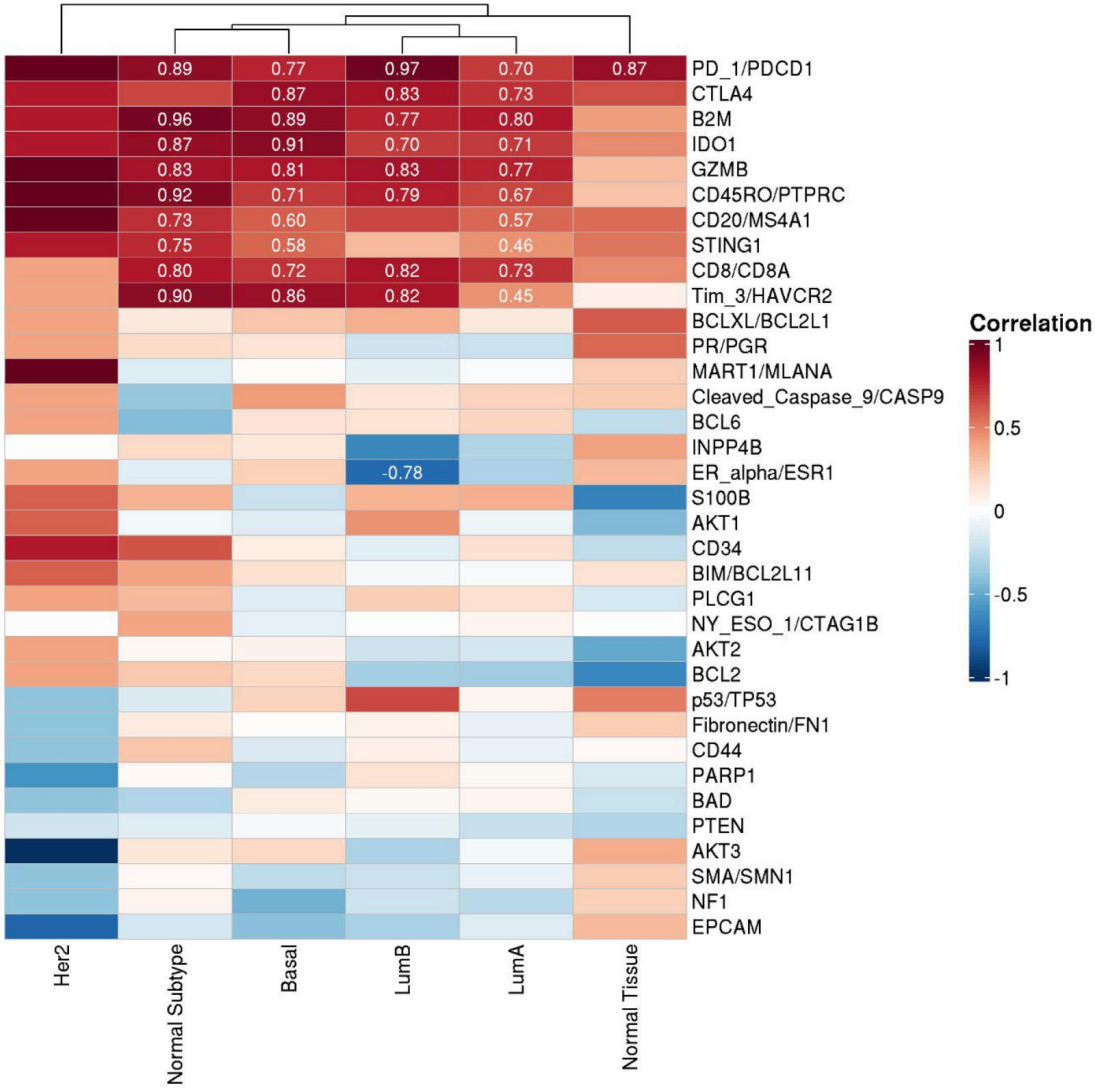
Correlation between TSLB-associated protein markers and a T-cell exhaustion signature in The Cancer Genome Atlas (TCGA) breast cancers diagnosed before age 40. Heatmap of Pearson correlation coefficients between log2-normalized RNA expression of genes corresponding to protein markers associated with time since last birth (TSLB) in our cohort and a previously published T-cell exhaustion (T_Ex_) signature score in TCGA breast cancer dataset. Analyses were restricted to tumors from women younger than 40 years (*n* = 79) and are shown by intrinsic molecular subtype (luminal A, luminal B, HER2-enriched, and triple-negative breast cancer). Multiple markers, including PD-1, CTLA4, B2M, IDO1, GZMB, CD45RO, CD20, STING1, CD8, and Tim-3, show strong positive correlations with T_Ex_ scores across subtypes, consistent with a coordinated immune-exhaustion program in early onset breast cancers exhibiting postpartum-associated protein signatures.

Among women less than age 40 years included in TCGA, only IDO1 was associated with age at BC diagnosis, after adjustment for multiple comparisons (data not shown). Multiple biomarkers in BCs associated with TSLB were correlated with a T-cell exhaustion signature across BC molecular subtypes (range: *r* = 0.45-0.96), including PD-1, CTLA4, B2M, IDO1, GZMB, CD45RO, CD20, STING1, CD8, and Tim3 (Figure 2). In normal tissues, none of the markers was associated with TSLB.

## Discussion

We hypothesize that defining specific associations between protein biomarkers and TSLB would support the hypothesis that PPBC represents a distinct phenotype. Using DSP, we identified consistent, significant associations between TSLB and multiple protein biomarkers in 640 BCs diagnosed at age ≤40; these associations persisted after adjustment for molecular subtype, are strongest with shortest TSLB, and persist with multiple testing correction. To replicate and contextualize our DSP findings, which were measured in 600-µm segmented ROIs, we applied OPAL multiplex immunofluorescence to quantify marker expression across entire TMA cores, using CK^+^ (epithelial) and CK^−^ (stromal) gates to mirror DSP keratin-rich and keratin-poor segmented compartments. We confirmed several DSP associations, particularly for PR and PTEN in CK^+^ epithelium when considering all women, and observed additional subtype-specific signals in parous women and all women, including nulliparas. Markers associated with TSLB also correlated with a T-cell exhaustion signature in TCGA, aligning with prior reports of T-cell signaling in early onset BCs of parous women.^22^

Our molecular epidemiological analysis complements findings reported by Jindal et al.,^22^ which used a bioinformatics approach to analyze bulk RNA sequencing from 16 ER-positive BCs in women <45 years. This study linked genes expressed in early onset BCs of parous women to those in postpartum remodeling and generated a prognostic signature. The prognostic signature, based on genes related to T-cell presence-activation/immune exhaustion, *E2F1*-mediated expression, *p53* and *ESR1*, predicted poor survival among BC patients aged ≤45 years in public datasets; however, detailed parity (TSLB) information was lacking in these data. In contrast, we assessed predefined protein markers in 640 BCs, including all intrinsic molecular subtypes, in relation to parity and TSLB, modeled continuously and in ordered categories, and adjusted for potential confounders. In our prospectively enrolled cohort, we used spatially resolved protein profiling (DSP) and OPAL multiplex immunofluorescence rather than bulk RNA-seq without microdissection. Notably, we confirm the reported associations of PPBC with T-cell signaling by showing that proteins associated with shorter TSLB, including CTLA4, PD-1, GZMB, and CD20, correlate strongly with a T-cell exhaustion transcriptomic score in TCGA, consistent with the well-recognized coupling of T-cell activation/presence and exhaustion under chronic antigenic stimulation. We also identify increasing PR levels (representing a readout of estradiol-ER mediated transcription) with increasing TSLB. Together, these complementary datasets support the existence of a postpartum-associated immune program.

In contrast to the prognostic signature reported by Jindal et al., we did not identify robust association of either p53 or ER-α levels with TSLB. Although Jindal et al. reported *TP53* mutations in some BCs among parous women, p53 immunostaining was not statistically different between postpartum and nonpostpartum BCs.^22^  *P53* mutations are found in 40%-60% of unselected BCs and in 85% of TNBCs.^40^ An analysis of 313 BCs found that *p53* mutations were not statistically significantly associated with parity; however, mutations in *P53* and *MYC* amplification were more frequent and *CDH1* mutations were less frequent among women with early age at first birth.^41^ Staining for p53 protein offers an imperfect surrogate of mutation and gene function.^42^ Analysis of *p53* signatures, as reported in Jindal et al.,^22^ may provide greater insights into loss (or gain) of *p53* functions, although such alterations are common in diverse types of BCs.^42^^,^^43^

Based on our data and other results, we suggest that some BCs that occur after a recent birth may exhibit a distinctive molecular profile, while recognizing that optimal diagnostic criteria would require consideration of complex interactions among biomarker levels, as revealed by SIR, and different temporal and spatial patterns of marker expression by BC subtype, which can be obscured when considering all BCs combined. While TSLB may be linked to poor prognosis, it is inconsistently recorded in clinical records. Encouraging clinicians to routinely document TSLB would facilitate research on PPBCs. In addition, because many PPBCs are associated with PTEN and PI3k/Akt pathway alterations, molecular testing may be indicated to determine eligibility for target therapies.

Data suggest that molecular signatures that distinguish parous from nulliparous normal breast tissues are mirrored in ER+ BCs of parous women,^22^ and such signatures may help distinguish PPBCs from other early onset BCs that develop via different mechanisms and might be amenable to specific targeted therapies (eg, *BRCA1* or *BRCA2* and poly [ADP-ribose] polymerase inhibitors).^44^ Accordingly, studies that pair molecular analyses with reproductive history data are needed to refine criteria for PPBC and potentially distinguish these BCs from other early onset subtypes. While all PPBCs are early onset, we hypothesize that not all early onset BCs among parous women are PPBCs, as defined by biomarker expression and mechanisms of pathogenesis.

Our analysis has potential relevance for discovering targeted therapies. SMA, a biomarker expressed in CAFs, is associated with increasing TSLB in HER2-enriched BCs and inversely associated in TNBCs. Experimental models suggest that CAFs produce estrogen, progesterone and growth factors that support anchorage independent growth, formation of tumorspheres and stemness.^45^^,^^46^ Here, SMA correlated with immune suppressive markers (within stroma), including immune checkpoints B7-H3 (structurally related to B7-H4, see “Introduction”) (*r* = 0.59), PD-1 (*r* = 0.61), and Tim-3 (*r* = 0.55). A meta-analysis found that increased SMA levels were associated with reduced BC recurrence-free survival.^47^ Conditioned media from short passaged CAFs with high SMA levels reportedly increase growth of MCF7 BC cells and elevate expression of podoplanin, a marker of lymphangiogenesis that is upregulated in PPBCs and co-expressed in CAFs.^48^ Studies link both SMA and podoplanin to resistance to trastuzumab in HER2+ BCs.^49^

Activation of the procarcinogenic PI3K/Akt/mTOR pathway is frequent in BC.^50^^,^^51^ Loss of PTEN tumor suppressor gene function may activate PI3K/Akt/mTOR and ER signaling and is linked to poor prognosis in triple-negative BCs and therapeutic resistance in ER-positive BCs.^50^^,^^51^ We identified higher PTEN levels with longer TSLB in luminal A and HER2+ BCs and lower levels with longer TSLB in triple-negative BCs, with some analyses also showing relationships with Akt pathway members, suggesting additional analyses of this pathway in PPBC may have value in translational research.

While supporting proof-of-concept that PPBC represents a distinctive BC type, defined by TSLB and marker expression, our study is limited by evaluating a prespecified set (albeit a large set) of candidate markers, technical challenges related to marker measurement, and limited racial and ethnic diversity of the participants. Molecular profiling, single cell RNA analysis, genetic germline testing and analyses of survival outcomes might extend the impact of this work. Nonetheless, our findings are robust when assessed with different statistical methods, replicated with a complementary laboratory method, and leveraged detailed epidemiological information. We conclude that PPBCs are distinguishable based on combinations of clinical features and molecular markers, and that defining criteria for this diagnosis may have value for prevention and therapy.

## Supplementary Material

djag003_Supplementary_Data

## Data Availability

Data will be provided upon reasonable request to the corresponding author. Associations of time since last birth (TSLB) and top biomarkers,^a^ and multivariate associations between time since last birth (TSLB) and the residuals,^b^ among 336 parous study participants with known TSLB^c^ in keratin-rich segments. Results for biomarkers with *P* ≤ .01 shown (see Supplementary Material for complete results). Bold represents significant at the Bonferroni corrected level (0.05/53). Abbreviations: BC = breast cancer; BMI = body mass index; ROI = region of interest; SIR = Sliced Inverse Regression; TSLB = time since last birth. Expression values of the 53 biomarkers were log2-transformed. Residuals from linear regressions of the 53 log-transformed biomarkers individually explained by age at diagnosis, number of live children prediagnosis, breastfeeding, menopausal status, family history of ovarian or BC, and BMI in the parous study participants with known TSLB. Thirty-six parous study participants with missing TSLB removed from the analytic dataset. Continuous TSLB used in linear regression models with each individual biomarker as outcome and adjusting for age at diagnosis, number of live children prediagnosis, breastfeeding, menopausal status, family history of ovarian or BC, and BMI in the parous study participants with known TSLB. Student *t*-tests were used to assess the significance of the associations. Wald-type tests testing the importance of each individual biomarker residual in all 3 SIR components obtained from the parous study participants with known TSLB. SIR components defined as weighted sums of the 53 biomarker residuals. Marginal dimension test indicated 3 SIR components were needed. Repeated ROIs of the 336 parous study participants with known TSLB considered as independent observations (*n* = 789).

## References

[1] Anderson WF , Rosenberg PS, Prat A, et al How many etiological subtypes of breast cancer: two, three, four, or more? J Natl Cancer Inst. 2014;106:1–11. 10.1093/jnci/dju165 PMC4148600 25118203

[2] Allott EH , Shan Y, Chen M, et al Bimodal age distribution at diagnosis in breast cancer persists across molecular and genomic classifications. Breast Cancer Res Treat. 2020;179:185-195. 31535320 10.1007/s10549-019-05442-2 PMC6985047

[3] Goldhirsch A , Gelber RD, Yothers G, et al Adjuvant therapy for very young women with breast cancer: need for tailored treatments. J Natl Cancer Inst Monogr. 2001;2001:44-51. 10.1093/oxfordjournals.jncimonographs.a003459(30):44-51 11773291

[4] Partridge AH , Hughes ME, Warner ET, et al Subtype-dependent relationship between young age at diagnosis and breast cancer survival. J Clin Oncol. 2016;34:3308-3314. 27480155 10.1200/JCO.2015.65.8013

[5] Nichols HB , Schoemaker MJ, Cai J, et al Breast cancer risk after recent childbirth: a pooled analysis of 15 prospective studies. Ann Intern Med. 2019;170:22-30. 30534999 10.7326/M18-1323 PMC6760671

[6] Mathews TJ , Hamilton BE. Mean age of mothers is on the rise: United States, 2000-2014. *NCHS Data Brief*. 2016;232:1–8. 26828319

[7] Gupta A , Akinyemiju T. Early-onset cancer incidence in the United States by race/ethnicity between 2011 and 2020. Cancer Epidemiol. 2024;92:102632. 39094298 10.1016/j.canep.2024.102632

[8] Borges VF , Lyons TR, Germain D, et al Postpartum involution and cancer: an opportunity for targeted breast cancer prevention and treatments? Cancer Res. 2020;80:1790-1798. 32075799 10.1158/0008-5472.CAN-19-3448 PMC8285071

[9] Jindal S , Gao D, Bell P, et al Postpartum breast involution reveals regression of secretory lobules mediated by tissue-remodeling. Breast Cancer Res. 2014;16:R31. 24678808 10.1186/bcr3633 PMC4053254

[10] Ogony J , de Bel T, Radisky DC, et al Towards defining morphologic parameters of normal parous and nulliparous breast tissues by artificial intelligence. Breast Cancer Res. 2022;24:45. 35821041 10.1186/s13058-022-01541-z PMC9275035

[11] Figueroa JD , Pfeiffer RM, Patel DA, et al Terminal duct lobular unit involution of the normal breast: implications for breast cancer etiology. J Natl Cancer Inst. 2014;106:1–11. 10.1093/jnci/dju286 PMC4200067 25274491

[12] Rotunno M , Sun X, Figueroa J, et al Parity-related molecular signatures and breast cancer subtypes by estrogen receptor status. Breast Cancer Res. 2014;16:R74. 25005139 10.1186/bcr3689 PMC4227137

[13] Asztalos S , Gann PH, Hayes MK, et al Gene expression patterns in the human breast after pregnancy. Cancer Prev Res (Phila). 2010;3:301-311. 20179293 10.1158/1940-6207.CAPR-09-0069

[14] Santucci-Pereira J , Zeleniuch-Jacquotte A, Afanasyeva Y, et al Genomic signature of parity in the breast of premenopausal women. Breast Cancer Res. 2019;21:46. 30922380 10.1186/s13058-019-1128-x PMC6438043

[15] Callihan EB , Gao D, Jindal S, et al Postpartum diagnosis demonstrates a high risk for metastasis and merits an expanded definition of pregnancy-associated breast cancer. Breast Cancer Res Treat. 2013;138:549-559. 23430224 10.1007/s10549-013-2437-x PMC3608871

[16] Goddard ET , Bassale S, Schedin T, et al Association between postpartum breast cancer diagnosis and metastasis and the clinical features underlying risk. JAMA Netw Open. 2019;2:e186997. 30646210 10.1001/jamanetworkopen.2018.6997 PMC6484560

[17] Lefrere H , Moore K, Floris G, et al Poor outcome in postpartum breast cancer patients is associated with distinct molecular and immunologic features. Clin Cancer Res. 2023;29:3729-3743. 37449970 10.1158/1078-0432.CCR-22-3645 PMC10502474

[18] Yu J , Yan Y, Li S, et al Progestogen-driven B7-H4 contributes to onco-fetal immune tolerance. Cell. 2024;187:4713-4732.e19. 38968937 10.1016/j.cell.2024.06.012 PMC11344674

[19] Phipps M , Falchook GS. B7 homolog 4 (B7-H4)-directed agents in oncology clinical trials: a review. J Immunother Precis Oncol. 2025;8:153-160. 40212845 10.36401/JIPO-24-34 PMC11985252

[20] Elia A , Saldain L, Vanzulli SI, et al Beneficial effects of mifepristone treatment in patients with breast cancer selected by the progesterone receptor isoform ratio: results from the MIPRA trial. Clin Cancer Res. 2023;29:866-877. 36269797 10.1158/1078-0432.CCR-22-2060 PMC9975668

[21] Egelston CA , Guo W, Tan J, et al Tumor-infiltrating exhausted CD8+ T cells dictate reduced survival in premenopausal estrogen receptor-positive breast cancer. JCI Insight. 2022;7:e153963. 10.1172/jci.insight.153963 PMC8855819 35132960

[22] Jindal S , Pennock ND, Sun D, et al Postpartum breast cancer has a distinct molecular profile that predicts poor outcomes. Nat Commun. 2021;12:6341. 34732713 10.1038/s41467-021-26505-3 PMC8566602

[23] Vohra SN , Walens A, Hamilton AM, et al Molecular and clinical characterization of postpartum-associated breast cancer in the Carolina Breast Cancer Study Phase I-III, 1993-2013. Cancer Epidemiol Biomarkers Prev. 2022;31:561-568. 34810211 10.1158/1055-9965.EPI-21-0940 PMC8901538

[24] Lyons TR , Borges VF, Betts CB, et al Cyclooxygenase-2-dependent lymphangiogenesis promotes nodal metastasis of postpartum breast cancer. J Clin Invest. 2014;124:3901-3912. 25133426 10.1172/JCI73777 PMC4153700

[25] Martinson HA , Jindal S, Durand-Rougely C, et al Wound healing-like immune program facilitates postpartum mammary gland involution and tumor progression. Int J Cancer. 2015;136:1803-1813. 25187059 10.1002/ijc.29181 PMC4324053

[26] Amant F , Lefrere H, Borges VF, et al The definition of pregnancy-associated breast cancer is outdated and should no longer be used. Lancet Oncol. 2021;22:753-754. 34087122 10.1016/S1470-2045(21)00183-2 PMC8868503

[27] Pena-Enriquez R , Bermejo B, Pollan M, et al Molecular characterization of pregnancy-associated breast cancer and insights on timing from GEICAM-EMBARCAM study. NPJ Breast Cancer. 2025;11:12. 39922815 10.1038/s41523-025-00718-x PMC11807221

[28] Hartman EK , Eslick GD. The prognosis of women diagnosed with breast cancer before, during and after pregnancy: a meta-analysis. Breast Cancer Res Treat. 2016;160:347-360. 27683280 10.1007/s10549-016-3989-3

[29] Lyons TR , O’Brien J, Borges VF, et al Postpartum mammary gland involution drives progression of ductal carcinoma in situ through collagen and COX-2. Nat Med. 2011;17:1109-1115. 21822285 10.1038/nm.2416 PMC3888478

[30] Lefrere H , Floris G, Schmidt MK, et al Breast cancer diagnosed in the post-weaning period is indicative for a poor outcome. Eur J Cancer. 2021;155:13-24. 34330022 10.1016/j.ejca.2021.06.009

[31] Rosenberg SM , Zheng Y, Ruddy K, et al Helping ourselves, helping others: the Young Women’s Breast Cancer Study (YWS)—a multisite prospective cohort study to advance the understanding of breast cancer diagnosed in women aged 40 years and younger. BMJ Open. 2024;14:e081157. 10.1136/bmjopen-2023-081157 PMC11218027 38951008

[32] Collins LC , Gelber S, Marotti JD, et al Molecular phenotype of breast cancer according to time since last pregnancy in a large cohort of young women. Oncologist. 2015;20:713-718. 26025931 10.1634/theoncologist.2014-0412 PMC4492229

[33] Bergholtz H , Carter JM, Cesano A, et al Best practices for spatial profiling for breast cancer research with the GeoMx((R)) digital spatial profiler. Cancers (Basel). 2021;13:1–27. 10.3390/cancers13174456 PMC8431590 34503266

[34] Li K-C. Sliced inverse regression of dimension reduction. JASA. 1991;86:316-327.

[35] Shao Y , Cook DR, Weisberg S. Marginal tests with sliced average variance estimation. Biometrika. 2007;94:285-296.

[36] Cook R. Testing predictor contributions in sufficient dimension reduction. Ann. Statist. 2004;32:1062-1092.

[37] Becht E , McInnes L, Healy J, et al Dimensionality reduction for visualizing single-cell data using UMAP. Nat Biotechnol. 2018;37:38–44. 10.1038/nbt.4314 30531897

[38] Love MI , Huber W, Anders S. Moderated estimation of fold change and dispersion for RNA-seq data with DESeq2. Genome Biol. 2014;15:550. 25516281 10.1186/s13059-014-0550-8 PMC4302049

[39] Benjamini Y , Hochber Y. Controlling the false discovery rate: a practical and powerful approach to multiple testing. J R Stat Soc Ser B. 1995;57:289-300.

[40] Pollock NC , Ramroop JR, Hampel H, et al Differences in somatic TP53 mutation type in breast tumors by race and receptor status. Breast Cancer Res Treat. 2022;192:639-648. 35286522 10.1007/s10549-022-06509-3 PMC8960361

[41] Nguyen B , Venet D, Lambertini M, et al Imprint of parity and age at first pregnancy on the genomic landscape of subsequent breast cancer. Breast Cancer Res. 2019;21:25. 30770770 10.1186/s13058-019-1111-6 PMC6377756

[42] Miller LD , Smeds J, George J, et al An expression signature for p53 status in human breast cancer predicts mutation status, transcriptional effects, and patient survival. Proc Natl Acad Sci U S A. 2005;102:13550-13555. 16141321 10.1073/pnas.0506230102 PMC1197273

[43] Marvalim C , Datta A, Lee SC. Role of p53 in breast cancer progression: an insight into p53 targeted therapy. Theranostics. 2023;13:1421-1442. 36923534 10.7150/thno.81847 PMC10008729

[44] Rezoug Z , Totten SP, Szlachtycz D, et al Universal genetic testing for newly diagnosed invasive breast cancer. JAMA Netw Open. 2024;7:e2431427. 39226054 10.1001/jamanetworkopen.2024.31427 PMC11372499

[45] Diep CH , Spartz A, Truong TH, et al Progesterone receptor signaling promotes cancer associated fibroblast mediated tumorigenicity in ER+ breast cancer. Endocrinology. 2024;165:1–17. 10.1210/endocr/bqae092 PMC11492492 39041201

[46] Wu F , Yang J, Liu J, et al Signaling pathways in cancer-associated fibroblasts and targeted therapy for cancer. Signal Transduct Target Ther. 2021;6:218. 34108441 10.1038/s41392-021-00641-0 PMC8190181

[47] Cui M , Dong H, Duan W, et al The relationship between cancer associated fibroblasts biomarkers and prognosis of breast cancer: a systematic review and meta-analysis. PeerJ. 2024;12:e16958. 38410801 10.7717/peerj.16958 PMC10896086

[48] Dahms P , Lyons TR. Toward characterizing lymphatic vasculature in the mammary gland during normal development and tumor-associated remodeling. J Mammary Gland Biol Neoplasia. 2024;29:1. 38218743 10.1007/s10911-023-09554-w PMC10787674

[49] Vathiotis IA , Moutafi MK, Divakar P, et al Alpha-smooth muscle actin expression in the stroma predicts resistance to trastuzumab in patients with early-stage HER2-positive breast cancer. Clin Cancer Res. 2021;27:6156-6163. 34465600 10.1158/1078-0432.CCR-21-2103 PMC8595766

[50] Chai C , Wu HH, Abuetabh Y, et al Regulation of the tumor suppressor PTEN in triple-negative breast cancer. Cancer Lett. 2022;527:41-48. 34902523 10.1016/j.canlet.2021.12.003

[51] Liu L , Graff SL, Wang Y. New emerging therapies targeting PI3K/AKT/mTOR/PTEN pathway in hormonal receptor-positive and HER2-negative breast cancer-current state and molecular pathology perspective. Cancers (Basel). 2024;17:1-12. 39796647 10.3390/cancers17010016 PMC11718791

