# Supplementary material for: Postpartum breast cancer: evidence for a distinct phenotype
Source: J Natl Cancer Inst. 2026 Jan 9;118(5):926–36. doi: 10.1093/jnci/djag003 (PMC13155230; doi:10.1093/jnci/djag003)
Supplement: djag003_Supplementary_Data [file djag003_supplementary_data.docx]

**Supplemental Methods**

NanoString Digital Spatial Profiling (DSP) Assay

Three negative control biomarkers (Rb-IgG, Ms-IgG2a, Ms-IgG1) and three housekeeping biomarkers (S6, Histone-H3, GAPDH) were used for quality control and then removed from the biomarker set prior to analysis [33]. Tag counts of the 71 remaining biomarkers were log2-transformed and checked for adequate quality, defined as log-expression greater than the log-expression of the 3 negative control biomarkers in at least 5% of observations, and average log-expression over all observations larger than the average of the mean log-expression of the 3 negative controls. After quality control, 53 biomarkers remained in keratin-rich segments and 48 biomarkers remained in keratin-poor segments.

Multiplex Immunofluorescent (OPAL) Assay

Prior to multiplexing, each primary antibody was validated on serial sections by conventional chromogenic immunohistochemistry and by single-plex immunofluorescence to confirm expected staining pattern and optimal dilution, first in reference tissues with known antigen abundance and then in normal breast. Optimized conditions were implemented in a six-color Opal protocol (Akoya Biosciences). Briefly, slides underwent sequential rounds of antigen retrieval, primary antibody incubation, HRP conjugated secondary amplification and Opal fluorophore deposition, with microwave stripping (AR6 or AR9, Akoya) between cycles. A test slide containing all five antibodies was used to fine tune marker order and Opal–antibody pairing and served as a positive control in every staining batch. An autofluorescence only slide received the full HRP cycle without Opal fluorophore and was included in spectral unmixing to characterize and isolate tissue autofluorescence.

The final panel was applied in the following sequence: CD20 (Cell Signaling 48750, 1:400, Opal 520), PTEN (Cell Signaling 9559, 1:200, Opal 570), PR (Cell Signaling 8757, 1:500, Opal 480), CTLA4 (Abcam ab237712 1:400, Opal 620), SMA (Dako M085101, 1:800, Opal 690) and cytokeratin (CK) AE1/AE3 (Dako M3515, 1:200, Opal 780). DAPI counter stain was applied in the final step.

Slides were imaged on a PhenoImager HT 2.0 platform (Akoya Biosciences) using manufacturer’s recommended exposure times, spectral libraries and autofocus settings. Multispectral images were unmixed with inForm 2.6; the autofluorescence library generated from the control slide was applied to all samples. Epithelial regions were delineated by cytokeratin signal, nuclei were segmented by DAPI and fine-tuned with assisting components, and marker positive cells were identified with phenotype thresholds derived from single-plex controls.

Following image QC, we excluded cores with processing artifacts and applied pre‑specified cellularity thresholds: for epithelial‑restricted markers, cores with <100 CK⁺ cells were excluded; for stromal/CK⁻ markers, cores with total cellularity <(mean −2 SD) were excluded. After QC, 1,237 cores remained evaluable. CK⁺ gating was based on the cytokeratin mask; CK⁻ compartments comprised the complement within each core.

Marker counts were log‑transformed. Associations with time since last birth (TSLB) were evaluated in (i) parous‑only models and (ii) models including all women, where nulliparous participants were treated as having the longest TSLB. For OPAL, TSLB was analyzed as ordered categories with a linear trend; covariates included age at diagnosis, menopausal status, and family history of breast/ovarian cancer, with stratification by molecular subtype where indicated. For combined nulliparous + parous tables using categorical TSLB, the 11–21‑year category was the reference level, and trend tests used an ordinal score with nulliparous coded as the largest value.

**Table S1**. Association between time since last birth and demographic and clinical characteristics in the 336 parous study participants with known time since last birth

|  | Individual models | | Model including stepwise selected demographic and clinical characteristics | |
| --- | --- | --- | --- | --- |
| Demographic or clinical characteristic | P-value from polytomous regression likelihood ratio test^b^ | P-value from linear regression likelihood ratio test^c^ | P-value from polytomous regression likelihood ratio test^d^ | P-value from linear regression likelihood ratio test^e^ |
| Age at diagnosis | **1.00E-12** | **1.00E-10** | **2.00E-12** | **1.00E-13** |
| Age at menarche | 0.21 | 0.64 |  |  |
| Number of pregnancies pre-diagnosis^f^ | **0.01** | 0.24 |  |  |
| Number of live children pre-diagnosis | **2.00E-04** | 0.25 | **2.00E-04** | **0.009** |
| Number of miscarriages pre-diagnosis | 0.47 | 0.38 |  |  |
| Number of stillbirths pre-diagnosis | 0.12 | 0.17 |  |  |
| Number of abortions | 0.39 | 0.35 |  |  |
| Breastfeeding status | **0.05** | **3.00E-03** | 0.06 | **2.00E-05** |
| Menopause status | 0.10 | 0.08 |  | 0.08 |
| Family history of ovarian or BC | **0.03** | 0.19 | 0.07 |  |
| Smoking status | 0.26 | 0.7 |  |  |
| Drinking status | 0.20 | 0.17 |  |  |
| BMI at diagnosis | 0.56 | 0.52 |  |  |
| Race | 0.48 | 0.88 |  |  |
| Ethnicity | 0.53 | 0.56 |  |  |
| BC subtype (5 categories)^g^ | 0.31 | 0.13 |  | 0.08 |
| BC subtype (4 categories)^h^ | 0.27 | 0.11 |  |  |

^a^  36 parous study participants with missing TSLB were removed from the analytic data set, leaving 336 women for analysis

^b^ Each characteristic used individually in a polytomous regression model with TSLB in categories as outcome in the parous study participants with known TSLB, and likelihood ratio tests performed with the null model

^c^ Each characteristic used individually in a linear regression model with continuous TSLB as outcome in the parous study participants with known TSLB, and likelihood ratio tests performed with the null model

^d^ Stepwise selected characteristics used jointly in a polytomous regression model with TSLB in categories as outcome in the parous study participants with known TSLB, and likelihood ratio tests performed with the nested models where each characteristic is removed one at a time

^e^ Stepwise selected characteristics used jointly in a linear regression model with continuous TSLB as outcome in the parous study participants with known TSLB, and likelihood ratio tests performed with the nested models where each characteristic is removed one at a time

^f^ Number of pregnancies more than 5 years prior to BC diagnosis (resp. number of pregnancies less than 5 years prior to BC diagnosis) was not utilized because 41% of the women with small (resp. 44% of the women with large) TSLB had missing values

^g^ BC subtype in 5 categories (Luminal A, Luminal B, TNBC, HER2, Missing^i^)

^h^ BC subtype in 4 categories (Luminal A, Luminal B, TNBC, HER2). Study participants with missing BC^i^ subtype are removed from the analyses using this characteristic

^i^ Of the 336 parous study participants with known TSLB, 21 had missing BC subtype information

Abbreviations: BC, breast cancer; BMI, body mass index; TSLB, time since last birth

**Table S2.** Associations of time since last birth (TSLB) and 53 biomarkers^a^, and multivariate associations between TSLB and the 53 biomarkers residuals^b^, among 336 parous study participants with known TSLB^c^ in keratin-rich segments

|  | Univariate associations | | Multivariate associations |
| --- | --- | --- | --- |
| Biomarker | Slope estimate from marginal linear regression^d^ | P-value from test of significance in linear regression^d^ | P-value from test of significance^e^ in the 3 SIR^f^ components |
| B7_H3 | 0.01 | 0.7 | 0.24 |
| BAD | 0.02 | 0.12 | 0.02 |
| Bcl_2 | 0.04 | 0.07 | 0.08 |
| BCL6 | 0.03 | 3.00E-03 | 0.64 |
| BCLXL | 0.02 | 0.04 | 4.00E-03 |
| Beta_2_microglobulin | 0.01 | 0.33 | 2.00E-03 |
| BIM | 0.01 | 0.56 | 0.08 |
| CD11c | 0.01 | 0.13 | 0.81 |
| CD127 | -0.01 | 0.58 | 0.08 |
| CD14 | 0.01 | 0.47 | 0.12 |
| CD20 | -0.03 | **1.00E-03** | 0.01 |
| CD25 | 0.01 | 0.38 | 0.39 |
| CD3 | 0.01 | 0.53 | 0.56 |
| CD34 | 0.04 | 0.03 | 1.00E-03 |
| CD4 | 2.00E-03 | 0.79 | 0.14 |
| CD44 | -0.01 | 0.62 | 0.17 |
| CD45 | 0.01 | 0.54 | 0.86 |
| CD45RO | 0.02 | 0.01 | 0.56 |
| CD56 | 0.01 | 0.37 | 0.35 |
| CD68 | 0.01 | 0.5 | 0.81 |
| CD8 | 0.02 | 0.03 | 0.19 |
| Cleaved_Caspase_9 | 0.01 | 0.29 | 0.17 |
| CTLA4 | -0.03 | 0.03 | 0.32 |
| EpCAM | -0.03 | 0.1 | 0.35 |
| ER-alpha | 0.1 | 2.00E-03 | 0.62 |
| Fibronectin | 0.06 | 3.00E-03 | 1.00E-03 |
| GZMB | 0.02 | 0.05 | **4.00E-04** |
| Her2 | -1.00E-03 | 0.98 | 0.08 |
| HLA_DR | 4.00E-03 | 0.84 | 0.58 |
| IDO1 | 0.04 | 0.03 | 0.01 |
| INPP4B | 0.02 | 0.21 | 3.00E-03 |
| Ki_67 | 1.00E-03 | 0.96 | 0.06 |
| MART1 | 0.02 | 0.08 | 2.00E-03 |
| MET | -0.01 | 0.4 | 0.19 |
| NF1 | -0.01 | 0.7 | **1.00E-03** |
| NY_ESO_1 | 0.02 | 0.32 | 0.54 |
| p53 | -0.02 | 0.45 | 0.42 |
| Pan_AKT | 0.03 | 0.03 | 0.01 |
| PanCk | 0.02 | 0.34 | 0.59 |
| PARP | 0.03 | 0.01 | 0.21 |
| PD_1 | 0.02 | 0.01 | 0.02 |
| PD_L2 | 2.00E-03 | 0.93 | 0.29 |
| Phospho_AKT1_S473 | 0.02 | 0.13 | 0.83 |
| Phospho_GSK3A_S21_GSK3B_S9 | 0.02 | 0.11 | 0.63 |
| Phospho_PRAS40_T246 | 0.03 | 0.11 | 0.1 |
| PhosphoGSK3B_S9 | 0.06 | 0.06 | 0.23 |
| PLCG1 | 2.00E-03 | 0.88 | 0.01 |
| PR | 0.12 | **1.00E-04** | 0.11 |
| PTEN | 0.05 | **1.00E-03** | 0.08 |
| S100B | 0.02 | 0.57 | 0.01 |
| SMA | 0.04 | 0.03 | **1.00E-04** |
| STING | 0.04 | 0.01 | 0.33 |
| Tim_3 | 0.03 | 0.01 | 0.01 |

^a^ Expression values of the 53 biomarkers were log2-transformed

^b^ Residuals from linear regressions of the 53 log-transformed biomarkers^a^ individually explained by age at diagnosis, number of live children pre-diagnosis, breastfeeding, menopausal status, family history of ovarian or BC, and BMI, in the parous study participants with known TSLB^c^

^c^  36 parous study participants with missing TSLB removed from the analytic data set

^d^ Continuous TSLB used in linear regression models with each individual biomarker as outcome and adjusting for age at diagnosis, number of live children pre-diagnosis, breastfeeding, menopausal status, family history of ovarian or BC, and BMI, in the parous study participants with known TSLB. Student tests were used to assess the significance of the associations

^e^ Wald-type tests testing the importance of each individual biomarker residual in all three SIR^f^ components obtained from the parous study participants with known TSLB

^f^ SIR components defined as weighted sums of the 53 biomarker residuals. Marginal dimension test indicated 3 SIR components were needed. Repeated ROIs of the 336 parous study participants with known TSLB considered as independent observations (n = 789).

Bold represents significant at the Bonferroni corrected level (0.05/53)

Abbreviations: BC, breast cancer; BMI, body mass index; ROI, region of interest; SIR, Sliced Inverse Regression; TSLB, time since last birth.

**Table S3.** Individual associations between time since last birth (TSLB) and the 48 biomarkers^a^, and multivariate associations between TSLB and the 48 biomarkers residuals^b^, in the 335 parous study participants with known TSLB^c^ , in keratin-poor segments

|  | Individual associations | | Multivariate associations |
| --- | --- | --- | --- |
| Biomarker | Slope estimate from marginal linear regression^d^ | P-value from test of significance in marginal linear regression^d^ | P-value from test of significance^e^ in the 2 SIR^f^ components |
| B7_H3 | 0.02 | 0.14 | 0.46 |
| BAD | 0.01 | 0.08 | 0.94 |
| Bcl2 | 0.04 | 3.00E-03 | 0.28 |
| BCL6 | 0.03 | 5.00E-03 | 0.84 |
| BCLXL | 0.02 | 0.05 | 0.78 |
| Beta_2_microglobulin | 3.00E-03 | 0.69 | 0.09 |
| BIM | 0.01 | 0.34 | 0.02 |
| CD11c | 0.01 | 0.27 | 0.56 |
| CD127 | 0.01 | 0.15 | 0.87 |
| CD14 | 0.01 | 0.69 | 0.33 |
| CD20 | -0.04 | **3.00E-04** | 0.04 |
| CD25 | 0.02 | 0.08 | 0.64 |
| CD3 | 0.01 | 0.66 | 0.55 |
| CD34 | 0.03 | 0.11 | 0.05 |
| CD4 | 1.00E-03 | 0.92 | 0.71 |
| CD44 | -4.00E-03 | 0.78 | 2.00E-03 |
| CD45 | 3.00E-03 | 0.84 | 0.66 |
| CD45RO | 0.01 | 0.26 | 0.76 |
| CD56 | 0.01 | 0.22 | 0.81 |
| CD68 | -0.01 | 0.61 | 0.89 |
| CD8 | 0.01 | 0.34 | 0.44 |
| Cleaved_Caspase_9 | 0.02 | 2.00E-03 | 0.63 |
| CTLA4 | -0.07 | **4.00E-05** | 0.07 |
| EpCAM | -0.02 | 0.48 | 0.38 |
| ER-alpha | 0.04 | 0.05 | 0.02 |
| Fibronectin | 0.05 | 0.01 | **3.00E-05** |
| GZMB | 0.03 | 0.09 | 0.14 |
| Her2 | 0.01 | 0.84 | 0.55 |
| HLA_DR | 0.01 | 0.46 | 0.88 |
| IDO1 | 0.02 | 0.19 | 0.44 |
| INPP4B | 0.01 | 0.24 | 0.41 |
| Ki67 | 0.01 | 0.34 | 0.69 |
| MET | 0.01 | 0.32 | 0.29 |
| NF1 | 0.05 | 0.01 | 0.03 |
| NY_ESO_1 | 0.02 | 0.04 | 0.02 |
| p53 | 0.01 | 0.68 | 0.02 |
| Pan_AKT | 0.02 | 0.03 | **1.00E-03** |
| PanCk | 0.01 | 0.46 | 0.08 |
| PARP | 0.03 | 5.00E-03 | 0.48 |
| PD_1 | 0.02 | 4.00E-03 | 0.93 |
| Phospho_AKT1_S473 | 0.02 | 0.01 | 0.99 |
| Phospho_PRAS40_T246 | 0.03 | 0.06 | 0.43 |
| PR | 0.06 | **2.00E-04** | 0.12 |
| PTEN | 0.04 | **1.00E-03** | 0.01 |
| S100B | 0.02 | 0.50 | 0.04 |
| SMA | 0.03 | 0.11 | 0.87 |
| STING | 0.03 | 0.02 | 0.89 |
| Tim_3 | 0.03 | 0.01 | 0.06 |

^a^ Expression values of the biomarkers were log2-transformed checked for adequate quality. In the stromal data set, five markers were additionally excluded, leaving 48 markers for analysis

^b^ Residuals from linear regressions of the 48 log-transformed biomarkers^a^ individually explained by age at diagnosis, number of live children pre-diagnosis, breastfeeding, menopause status, family history of ovarian or BC, and BMI, in the parous study participants with known TSLB^c^

^c^  36 parous study participants with missing TSLB removed from the analytic data set

^d^ Continuous TSLB used in linear regression models with each individual biomarker as outcome and adjusting for age at diagnosis, number of live children pre-diagnosis, breastfeeding, menopause status, family history of ovarian or BC, and BMI, in the parous study participants with known TSLB. Student tests were used to assess the significance of the associations

^e^ Wald-type tests testing the importance of each individual biomarker residual in all three SIR^f^ components obtained from the parous study participants with known TSLB

^f^ SIR components defined as weighted sums of the 48 biomarker residuals. Marginal dimension test indicated 2 SIR components were needed. Repeated ROIs of the 335 parous study participants with known TSLB considered as independent observations (n = 790).

Bold reflects significant at the Bonferroni corrected level (0.05/48)

Abbreviations: BC, breast cancer; BMI, body mass index; ROI, region of interest; SIR, Sliced Inverse Regression; TSLB, time since last birth

**Table S4 A.** Results of multiplex immunofluorescent stains versus time since last birth among parous women

| Breast Cancer Subtype | **CTLA4 CD20**  **PR** **PTEN** **SMA______**  Est. (95%CI) p-value Est. (95%CI) p-value Est. (95%CI) p-value Est. (95%CI) p-value Est. (95%CI) p-value | | | | | | | | | |
| --- | --- | --- | --- | --- | --- | --- | --- | --- | --- | --- |
| Luminal A | 0.03  (-0.11-0.17) | 0.65 | 0.05  (-0.16-0.26) | 0.65 | 0.09  (-0.39-0.56) | 0.72 | 0.23  (-0.23-0.69) | 0.32 | 0.07  (-0.09-0.23) | 0.37 |
| Luminal B | 0.08  (-0.01-0.16) | 0.07 | 0.30  (-0.06-0.65) | 0.10 | -0.04  (-0.38--0.30) | 0.81 | -0.10  (-0.51-0.31) | 0.63 | 0.00  (-0.17-0.16) | 0.99 |
| HER2 | -0.05  (-0.24-0.14) | 0.60 | -0.14  ( -0.94-0.67) | 0.74 | -0.89  (-1.24- -0.53) | <0.001 | 0.72  (-0.10-1.55) | 0.09 | 0.18  (-0.10-0.45) | 0.20 |
| TNBC | 0.09  (-0.09-0.26) | 0.32 | -0.45  (-1.07-0.170 | 0.16 | 0.92  (0.21—1.62) | 0.01 | 0.04  (-0.66-0.75) | 0.90 | -0.29  (-0.53- -0.04 | 0.02 |

Linear regression models were adjusted for age at diagnosis, menopausal status, family history of ovarian or BC, and BMI

**Table S4 B.** Results of multiplex immunofluorescent stains versus time since last birth among all women

| Breast Cancer Subtype | **CTLA4 CD20**  **PR** **PTEN** **SMA______**  Est. (95%CI) p-value Est. (95%CI) p-value Est. (95%CI) p-value Est. (95%CI) p-value Est. | | | | | | | | | |
| --- | --- | --- | --- | --- | --- | --- | --- | --- | --- | --- |
| Luminal A | -0.01  (-0.06-.05) | 0.81 | 0.00  (-0.11-.11) | 0.99 | 0.23  (0.03-0.43) | 0.03 | 0.28  (0.10-0.46) | <0.01 | -0.02  (-0.10- 0.05) | 0.54 |
| Luminal B | 0.01  (-0.03-.05) | 0.51 | 0.01  (-0.12-.15) | 0.84 | 0.06  (-0.12-.25) | 0.49 | 0.11  (-0.09-0.31) | 0.28 | 0.04  (-0.04-0.12) | 0.33 |
| HER2 | -0.01  (-0.10-.09) | 0.55 | 0.03  (-0.43-.49) | 0.89 | 0.10  (-0.23- -0.42) | 0.89 | 0.45  (0.14-0.76) | 0.01 | 0.17  (0.05-0.28) | <0.01 |
| TNBC | 0.00  (-0.08--07) | 0.93 | -0.53  (-0.81- -.25 | <0.001 | -0.19  (-0.63-.24) | 0.38 | -0.62  (-0.96- -0.27) | <0.01 | -0.22  (-0.43-0.00) | 0.05 |

Linear regression models adjusted for age at diagnosis, menopausal status, family history of ovarian or BC, and BMI

**Table S5.** Demographic and clinical characteristics of 268 nulliparous women

**
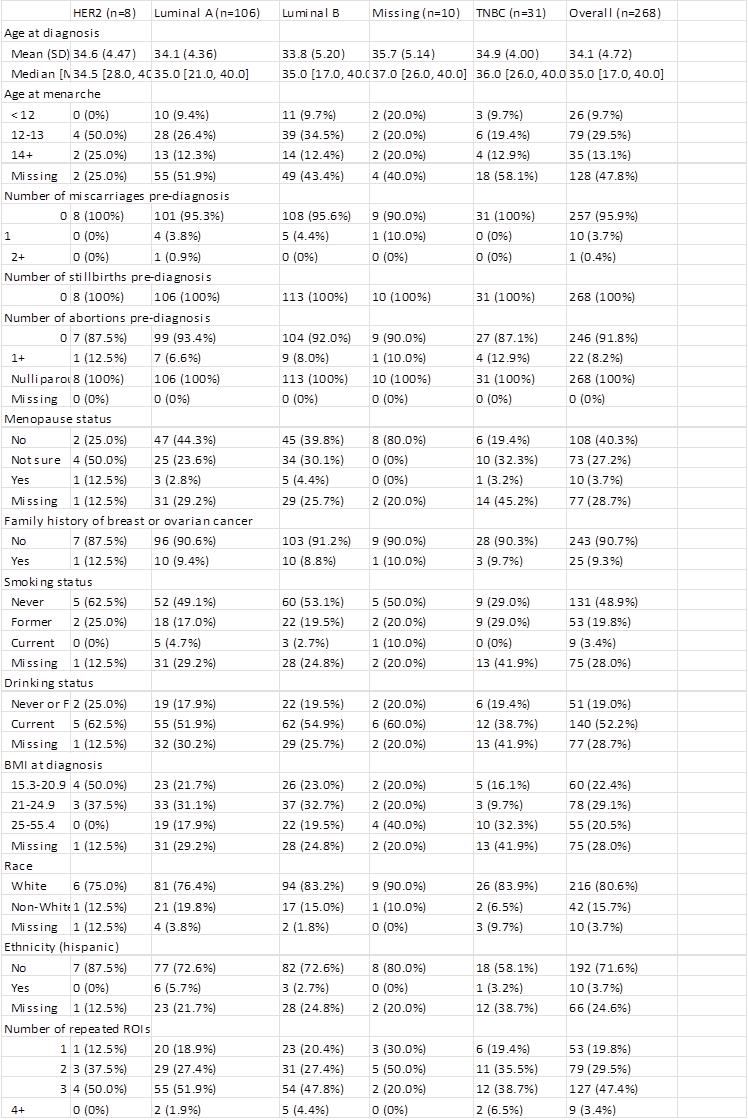
**

**Table S6**. Individual associations between time since last birth and the 53 biomarkers^a^, and multivariate associations between time since last birth and the 53 biomarkers residuals^b^, in the 604 nulliparous and parous study participants^c^

| \|  \| Individual associations \| \| \| \| \| \| \| --- \| --- \| --- \| --- \| --- \| --- \| --- \| \|  \| linear regression^d^ \| \| \| \| \| \| \|  \| TSLB in categories \| \| \| \| \| Continuous  TSLB^e^ \| \|  \| 0-3 years \| 3-5 years \| 6-10 years \| 11-21 years \| Nulliparous \| \| B7_H3 slope estimate \| -0.26 \| -0.29 \| -0.37 \| reference \| -0.97 \| -0.01 \| \| B7_H3 P-value \| 0.34 \| 0.31 \| 0.20 \| 0.42 \| 0.93 \| \| BAD slope estimate \| -0.18 \| -0.15 \| -0.12 \| reference \| 0.46 \| 0.06 \| \| BAD P-value \| 0.30 \| 0.39 \| 0.50 \| 0.07 \| 0.16 \| \| Bcl_2 slope estimate \| -0.39 \| -0.01 \| 0.05 \| reference \| 2.35 \| 0.23 \| \| Bcl_2 P-value \| 0.31 \| 0.98 \| 0.90 \| 0.01 \| 0.03 \| \| BCL6 slope estimate \| -0.34 \| -0.22 \| -0.17 \| reference \| 0.65 \| 0.11 \| \| BCL6 P-value \| 0.02 \| 0.14 \| 0.24 \| 0.03 \| 3.00E-03 \| \| BCLXL slope estimate \| -0.17 \| 0.04 \| 0.07 \| reference \| 0.57 \| 0.10 \| \| BCLXL P-value \| 0.35 \| 0.82 \| 0.72 \| 0.07 \| 0.02 \| \| Beta_2_microglobulin slope estimate \| -0.20 \| -0.30 \| -0.22 \| reference \| 0.47 \| 0.04 \| \| Beta_2_microglobulin P-value \| 0.30 \| 0.12 \| 0.27 \| 0.06 \| 0.35 \| \| BIM slope estimate \| -0.12 \| 0.10 \| -0.06 \| reference \| 1.46 \| 0.07 \| \| BIM P-value \| 0.44 \| 0.53 \| 0.68 \| 3.00E-07 \| 0.17 \| \| CD11c slope estimate \| -0.10 \| -0.13 \| -0.02 \| reference \| 0.27 \| 0.04 \| \| CD11c P-value \| 0.43 \| 0.34 \| 0.90 \| 0.12 \| 0.17 \| \| CD127 slope estimate \| 2.00E-03 \| 0.13 \| 0.01 \| reference \| 0.76 \| 0.02 \| \| CD127 P-value \| 0.99 \| 0.42 \| 0.96 \| 3.00E-04 \| 0.66 \| \| CD14 slope estimate \| -0.11 \| -0.16 \| -0.15 \| reference \| 0.51 \| 0.02 \| \| CD14 P-value \| 0.57 \| 0.44 \| 0.47 \| 0.19 \| 0.72 \| \| CD20 slope estimate \| 0.26 \| 0.16 \| 0.01 \| reference \| -0.17 \| -0.11 \| \| CD20 P-value \| 0.03 \| 0.18 \| 0.93 \| 0.62 \| **1.00E-03** \| \| CD25 slope estimate \| -0.22 \| -0.16 \| -0.21 \| reference \| 0.63 \| 0.05 \| \| CD25 P-value \| 0.16 \| 0.35 \| 0.19 \| 0.01 \| 0.18 \| \| CD3 slope estimate \| -0.02 \| -0.13 \| -0.06 \| reference \| 0.23 \| -4.00E-03 \| \| CD3 P-value \| 0.94 \| 0.48 \| 0.76 \| 0.28 \| 0.93 \| \| CD34 slope estimate \| -0.49 \| -0.28 \| -0.44 \| reference \| -0.12 \| 0.09 \| \| CD34 P-value \| 0.05 \| 0.28 \| 0.07 \| 0.75 \| 0.11 \| \| CD4 slope estimate \| -1.00E-03 \| -0.10 \| -0.02 \| reference \| 0.09 \| -3.00E-03 \| \| CD4 P-value \| 0.99 \| 0.37 \| 0.88 \| 0.55 \| 0.92 \| \| CD44 slope estimate \| -0.03 \| -0.38 \| -0.47 \| reference \| 0.62 \| -0.09 \| \| CD44 P-value \| 0.94 \| 0.39 \| 0.30 \| 0.45 \| 0.35 \| \| CD45 slope estimate \| -0.01 \| -0.11 \| -0.01 \| reference \| 0.45 \| 0.01 \| \| CD45 P-value \| 0.97 \| 0.61 \| 0.95 \| 0.07 \| 0.85 \| \| CD45RO slope estimate \| -0.26 \| -0.17 \| -0.13 \| reference \| 0.14 \| 0.08 \| \| CD45RO P-value \| 0.06 \| 0.23 \| 0.34 \| 0.56 \| 0.02 \| \| CD56 slope estimate \| -0.03 \| -0.08 \| 0.07 \| reference \| -0.08 \| 0.02 \| \| CD56 P-value \| 0.76 \| 0.39 \| 0.48 \| 0.51 \| 0.30 \| \| CD68 slope estimate \| -0.05 \| -0.12 \| -0.02 \| reference \| 0.27 \| 0.02 \| \| CD68 P-value \| 0.72 \| 0.34 \| 0.90 \| 0.09 \| 0.52 \| \| CD8 slope estimate \| -0.27 \| -0.33 \| -0.11 \| reference \| 0.28 \| 0.09 \| \| CD8 P-value \| 0.18 \| 0.08 \| 0.57 \| 0.23 \| 0.05 \| \| Cleaved_Caspase_9 slope estimate \| -0.08 \| -0.14 \| -0.05 \| reference \| -0.01 \| 0.02 \| \| Cleaved_Caspase_9 P-value \| 0.58 \| 0.36 \| 0.75 \| 0.99 \| 0.60 \| \| CTLA4 slope estimate \| 0.25 \| 0.22 \| -0.28 \| reference \| -1.14 \| -0.19 \| \| CTLA4 P-value \| 0.23 \| 0.31 \| 0.16 \| 0.06 \| **1.00E-03** \| \| EpCAM slope estimate \| 0.09 \| 0.05 \| -0.26 \| reference \| -0.21 \| -0.10 \| \| EpCAM P-value \| 0.77 \| 0.87 \| 0.43 \| 0.81 \| 0.21 \| \| ER_alpha slope estimate \| -1.01 \| -0.33 \| -0.51 \| reference \| 2.57 \| 0.35 \| \| ER_alpha P-value \| 0.08 \| 0.58 \| 0.41 \| 0.02 \| 0.02 \| \| Fibronectin slope estimate \| -0.49 \| -0.22 \| -0.08 \| reference \| -0.08 \| 0.18 \| \| Fibronectin P-value \| 0.15 \| 0.53 \| 0.81 \| 0.83 \| 0.02 \| \| GZMB slope estimate \| -0.30 \| -0.33 \| -0.31 \| reference \| -0.16 \| 0.04 \| \| GZMB P-value \| 0.07 \| 0.05 \| 0.07 \| 0.57 \| 0.24 \| \| Her2 slope estimate \| -0.08 \| 0.05 \| 0.15 \| reference \| 3.88 \| 0.16 \| \| Her2 P-value \| 0.92 \| 0.95 \| 0.85 \| 1.00E-04 \| 0.35 \| \| HLA_DR slope estimate \| -0.01 \| -0.23 \| -0.09 \| reference \| 0.69 \| -3.00E-03 \| \| HLA_DR P-value \| 0.97 \| 0.49 \| 0.80 \| 0.11 \| 0.97 \| \| IDO1 slope estimate \| -0.36 \| -0.44 \| -0.04 \| reference \| 0.78 \| 0.15 \| \| IDO1 P-value \| 0.31 \| 0.19 \| 0.92 \| 0.07 \| 0.06 \| \| INPP4B slope estimate \| -0.19 \| -0.01 \| 0.13 \| reference \| 1.62 \| 0.15 \| \| INPP4B P-value \| 0.53 \| 0.96 \| 0.67 \| 1.00E-05 \| 0.05 \| \| Ki_67 slope estimate \| -0.09 \| -0.11 \| -0.18 \| reference \| 0.11 \| -0.01 \| \| Ki_67 P-value \| 0.80 \| 0.75 \| 0.60 \| 0.79 \| 0.95 \| \| MART1 slope estimate \| -0.26 \| -0.28 \| -0.20 \| reference \| 0.87 \| 0.07 \| \| MART1 P-value \| 0.11 \| 0.09 \| 0.24 \| 1.00E-05 \| 0.08 \| \| MET slope estimate \| 0.13 \| 0.05 \| 0.16 \| reference \| -0.34 \| -0.02 \| \| MET P-value \| 0.52 \| 0.80 \| 0.44 \| 0.70 \| 0.71 \| \| NF1 slope estimate \| 0.31 \| 0.18 \| 0.43 \| reference \| -0.34 \| -0.03 \| \| NF1 P-value \| 0.41 \| 0.63 \| 0.28 \| 0.43 \| 0.72 \| \| NY_ESO_1 slope estimate \| -0.15 \| -0.32 \| -0.22 \| reference \| 0.60 \| 0.02 \| \| NY_ESO_1 P-value \| 0.47 \| 0.09 \| 0.30 \| 0.07 \| 0.73 \| \| p53 slope estimate \| 0.13 \| 0.05 \| 0.10 \| reference \| 1.83 \| 0.01 \| \| p53 P-value \| 0.73 \| 0.90 \| 0.81 \| 0.01 \| 0.92 \| \| Pan_AKT slope estimate \| -0.29 \| -0.02 \| 0.03 \| reference \| 0.62 \| 0.14 \| \| Pan_AKT P-value \| 0.08 \| 0.89 \| 0.85 \| 0.13 \| 2.00E-03 \| \| PanCk slope estimate \| -0.28 \| -0.18 \| -0.11 \| reference \| 2.72 \| 0.15 \| \| PanCk P-value \| 0.42 \| 0.60 \| 0.76 \| 1.00E-05 \| 0.12 \| \| PARP slope estimate \| -0.13 \| -2.00E-03 \| 0.14 \| reference \| 0.64 \| 0.10 \| \| PARP P-value \| 0.51 \| 0.99 \| 0.48 \| 0.02 \| 0.04 \| \| PD_1 slope estimate \| -0.17 \| -0.12 \| 0.10 \| reference \| 0.27 \| 0.10 \| \| PD_1 P-value \| 0.17 \| 0.31 \| 0.39 \| 0.05 \| **1.00E-03** \| \| PD_L2 slope estimate \| -0.12 \| 0.31 \| -0.04 \| reference \| 1.27 \| 0.07 \| \| PD_L2 P-value \| 0.69 \| 0.34 \| 0.91 \| 0.05 \| 0.34 \| \| Phospho_AKT1_S473 slope estimate \| -0.18 \| -0.08 \| -0.02 \| reference \| 0.17 \| 0.07 \| \| Phospho_AKT1_S473 P-value \| 0.34 \| 0.67 \| 0.90 \| 0.49 \| 0.16 \| \| Phospho_GSK3A_S21_GSK3B_S9 slope estimate \| -0.14 \| -0.16 \| -0.03 \| reference \| 0.37 \| 0.06 \| \| Phospho_GSK3A_S21_GSK3B_S9 P-value \| 0.32 \| 0.25 \| 0.82 \| 0.34 \| 0.14 \| \| Phospho_PRAS40_T246 slope estimate \| -0.17 \| -0.03 \| 0.01 \| reference \| -0.37 \| 0.06 \| \| Phospho_PRAS40_T246 P-value \| 0.63 \| 0.93 \| 0.98 \| 0.54 \| 0.41 \| \| PhosphoGSK3B_S9 slope estimate \| -0.65 \| -0.40 \| -0.06 \| reference \| 2.25 \| 0.30 \| \| PhosphoGSK3B_S9 P-value \| 0.18 \| 0.40 \| 0.91 \| 2.00E-04 \| 0.01 \| \| PLCG1 slope estimate \| -0.01 \| -0.08 \| 0.08 \| reference \| 0.47 \| 0.03 \| \| PLCG1 P-value \| 0.95 \| 0.69 \| 0.67 \| 0.01 \| 0.46 \| \| PR slope estimate \| -1.39 \| -0.99 \| -0.53 \| reference \| 3.64 \| 0.52 \| \| PR P-value \| 0.01 \| 0.06 \| 0.36 \| 3.00E-06 \| **1.00E-04** \| \| PTEN slope estimate \| -0.69 \| -0.53 \| -0.35 \| reference \| 1.13 \| 0.22 \| \| PTEN P-value \| 3.00E-03 \| 0.02 \| 0.14 \| 1.00E-03 \| **5.00E-04** \| \| S100B slope estimate \| 0.11 \| 0.20 \| 0.25 \| reference \| -1.09 \| -0.01 \| \| S100B P-value \| 0.78 \| 0.62 \| 0.55 \| 0.10 \| 0.93 \| \| SMA slope estimate \| -0.62 \| -0.57 \| -0.37 \| reference \| -2.02 \| 0.12 \| \| SMA P-value \| 0.03 \| 0.03 \| 0.18 \| 3.00E-10 \| 0.14 \| \| STING slope estimate \| -0.41 \| -0.22 \| -0.19 \| reference \| 0.57 \| 0.14 \| \| STING P-value \| 0.10 \| 0.40 \| 0.47 \| 0.11 \| 0.01 \| \| Tim_3 slope estimate \| -0.53 \| -0.42 \| -0.49 \| reference \| 0.84 \| 0.12 \| \| Tim_3 P-value \| 0.02 \| 0.08 \| 0.04 \| 0.03 \| 0.04 \| | \| Multivariate associations \| \| --- \| \| P-value from test of significance^f^ in the 3 SIR^g^ components \| \| 0.41 \| \| 0.03 \| \| 0.14 \| \| 0.83 \| \| 0.01 \| \| 0.02 \| \| 0.09 \| \| 0.87 \| \| 0.23 \| \| 0.41 \| \| 0.05 \| \| 0.08 \| \| 0.64 \| \| 0.01 \| \| 0.12 \| \| 0.43 \| \| 0.42 \| \| 0.47 \| \| 0.32 \| \| 0.97 \| \| 0.14 \| \| 0.37 \| \| 0.42 \| \| 0.24 \| \| 0.53 \| \| 0.01 \| \| 2.00E-04 \| \| 0.12 \| \| 0.69 \| \| 0.05 \| \| 0.04 \| \| 0.07 \| \| 0.01 \| \| 0.29 \| \| 0.01 \| \| 0.19 \| \| 0.61 \| \| 0.03 \| \| 0.55 \| \| 0.23 \| \| 0.05 \| \| 0.24 \| \| 0.98 \| \| 0.72 \| \| 0.49 \| \| 0.69 \| \| 0.01 \| \| 0.11 \| \| 0.19 \| \| 0.01 \| \| **1.00E-03** \| \| 0.36 \| \| 0.23 \| |
| --- | --- | --- | --- | --- | --- | --- | --- | --- | --- | --- | --- | --- | --- | --- | --- | --- | --- | --- | --- | --- | --- | --- | --- | --- | --- | --- | --- | --- | --- | --- | --- | --- | --- | --- | --- | --- | --- | --- | --- | --- | --- | --- | --- | --- | --- | --- | --- | --- | --- | --- | --- | --- | --- | --- | --- | --- | --- | --- | --- | --- | --- | --- | --- | --- | --- | --- | --- | --- | --- | --- | --- | --- | --- | --- | --- | --- | --- | --- | --- | --- | --- | --- | --- | --- | --- | --- | --- | --- | --- | --- | --- | --- | --- | --- | --- | --- | --- | --- | --- | --- | --- | --- | --- | --- | --- | --- | --- | --- | --- | --- | --- | --- | --- | --- | --- | --- | --- | --- | --- | --- | --- | --- | --- | --- | --- | --- | --- | --- | --- | --- | --- | --- | --- | --- | --- | --- | --- | --- | --- | --- | --- | --- | --- | --- | --- | --- | --- | --- | --- | --- | --- | --- | --- | --- | --- | --- | --- | --- | --- | --- | --- | --- | --- | --- | --- | --- | --- | --- | --- | --- | --- | --- | --- | --- | --- | --- | --- | --- | --- | --- | --- | --- | --- | --- | --- | --- | --- | --- | --- | --- | --- | --- | --- | --- | --- | --- | --- | --- | --- | --- | --- | --- | --- | --- | --- | --- | --- | --- | --- | --- | --- | --- | --- | --- | --- | --- | --- | --- | --- | --- | --- | --- | --- | --- | --- | --- | --- | --- | --- | --- | --- | --- | --- | --- | --- | --- | --- | --- | --- | --- | --- | --- | --- | --- | --- | --- | --- | --- | --- | --- | --- | --- | --- | --- | --- | --- | --- | --- | --- | --- | --- | --- | --- | --- | --- | --- | --- | --- | --- | --- | --- | --- | --- | --- | --- | --- | --- | --- | --- | --- | --- | --- | --- | --- | --- | --- | --- | --- | --- | --- | --- | --- | --- | --- | --- | --- | --- | --- | --- | --- | --- | --- | --- | --- | --- | --- | --- | --- | --- | --- | --- | --- | --- | --- | --- | --- | --- | --- | --- | --- | --- | --- | --- | --- | --- | --- | --- | --- | --- | --- | --- | --- | --- | --- | --- | --- | --- | --- | --- | --- | --- | --- | --- | --- | --- | --- | --- | --- | --- | --- | --- | --- | --- | --- | --- | --- | --- | --- | --- | --- | --- | --- | --- | --- | --- | --- | --- | --- | --- | --- | --- | --- | --- | --- | --- | --- | --- | --- | --- | --- | --- | --- | --- | --- | --- | --- | --- | --- | --- | --- | --- | --- | --- | --- | --- | --- | --- | --- | --- | --- | --- | --- | --- | --- | --- | --- | --- | --- | --- | --- | --- | --- | --- | --- | --- | --- | --- | --- | --- | --- | --- | --- | --- | --- | --- | --- | --- | --- | --- | --- | --- | --- | --- | --- | --- | --- | --- | --- | --- | --- | --- | --- | --- | --- | --- | --- | --- | --- | --- | --- | --- | --- | --- | --- | --- | --- | --- | --- | --- | --- | --- | --- | --- | --- | --- | --- | --- | --- | --- | --- | --- | --- | --- | --- | --- | --- | --- | --- | --- | --- | --- | --- | --- | --- | --- | --- | --- | --- | --- | --- | --- | --- | --- | --- | --- | --- | --- | --- | --- | --- | --- | --- | --- | --- | --- | --- | --- | --- | --- | --- | --- | --- | --- | --- | --- | --- | --- | --- | --- | --- | --- | --- | --- | --- | --- | --- | --- | --- | --- | --- | --- | --- | --- | --- | --- | --- | --- | --- | --- | --- | --- | --- | --- | --- | --- | --- | --- | --- | --- | --- | --- | --- | --- | --- | --- | --- | --- | --- | --- | --- | --- | --- | --- | --- | --- | --- | --- | --- | --- | --- | --- | --- | --- | --- | --- | --- | --- | --- | --- | --- | --- | --- | --- | --- | --- | --- | --- | --- | --- | --- | --- | --- | --- | --- | --- | --- | --- | --- | --- | --- | --- | --- | --- | --- | --- | --- | --- | --- | --- | --- | --- | --- | --- | --- | --- | --- | --- | --- | --- | --- | --- | --- | --- | --- | --- | --- | --- | --- | --- | --- | --- | --- | --- | --- | --- | --- | --- | --- | --- | --- | --- | --- | --- | --- | --- | --- | --- | --- | --- | --- | --- | --- | --- | --- | --- | --- | --- | --- | --- | --- | --- | --- | --- | --- | --- | --- | --- | --- | --- | --- | --- | --- | --- | --- | --- | --- | --- | --- | --- | --- | --- | --- | --- | --- | --- | --- | --- | --- | --- | --- | --- | --- | --- | --- | --- | --- | --- | --- | --- | --- | --- | --- | --- | --- | --- | --- | --- | --- | --- | --- | --- | --- | --- | --- | --- | --- | --- | --- | --- | --- | --- | --- | --- | --- | --- | --- | --- | --- | --- | --- | --- | --- | --- | --- | --- | --- | --- | --- | --- | --- | --- | --- | --- | --- | --- | --- | --- | --- | --- | --- | --- | --- | --- | --- | --- | --- | --- | --- | --- | --- | --- | --- | --- | --- | --- | --- | --- | --- | --- | --- | --- | --- |

^a^ Expression values of the 53 biomarkers were log2-transformed

^b^ Residuals from linear regressions of the 53 log-transformed biomarkers^a^ individually explained by age at diagnosis, number of live children pre-diagnosis, breastfeeding, menopause status, family history of ovarian or BC, and BMI, in the nulliparous and parous study participants with known TSLB^c^

^c^  36 parous study participants with missing TSLB removed from the analytic data set. Categorical version of TSLB includes nulliparous as a separate group

^d^ Categorical TSLB^c^ used in linear regression models with each individual biomarker as outcome and adjusting for age at diagnosis, number of live children pre-diagnosis, breastfeeding, menopause status, family history of ovarian or BC, and BMI, in the nulliparous and parous study participants with known TSLB. “11-21 years” was used as the TSLB reference category. Student tests were used to assess the significance of the associations

^e^ Trends across categories assessed by using an ordinal version of TSLB, with nulliparous as a separate group assumed to have the largest value, and treating it as a continuous variable in the models

^f^ Wald-type tests testing the importance of each individual biomarker residual in all three SIR^g^ components obtained from the nulliparous^c^ and parous study participants with known TSLB

^g^ SIR components defined as weighted sums of the 53 biomarker residuals. Marginal dimension test indicated 3 SIR components were needed. Repeated ROIs of the 604 study participants considered as independent observations (n = 1424).

**^*^** Significant at the Bonferroni corrected level (0.05/53)

**Figure S1 A. Heatmap of reproductive risk factor levels in relation to biomarker levels in keratin-rich segments of ROIs of 336 parous women ages < 40 years**

**
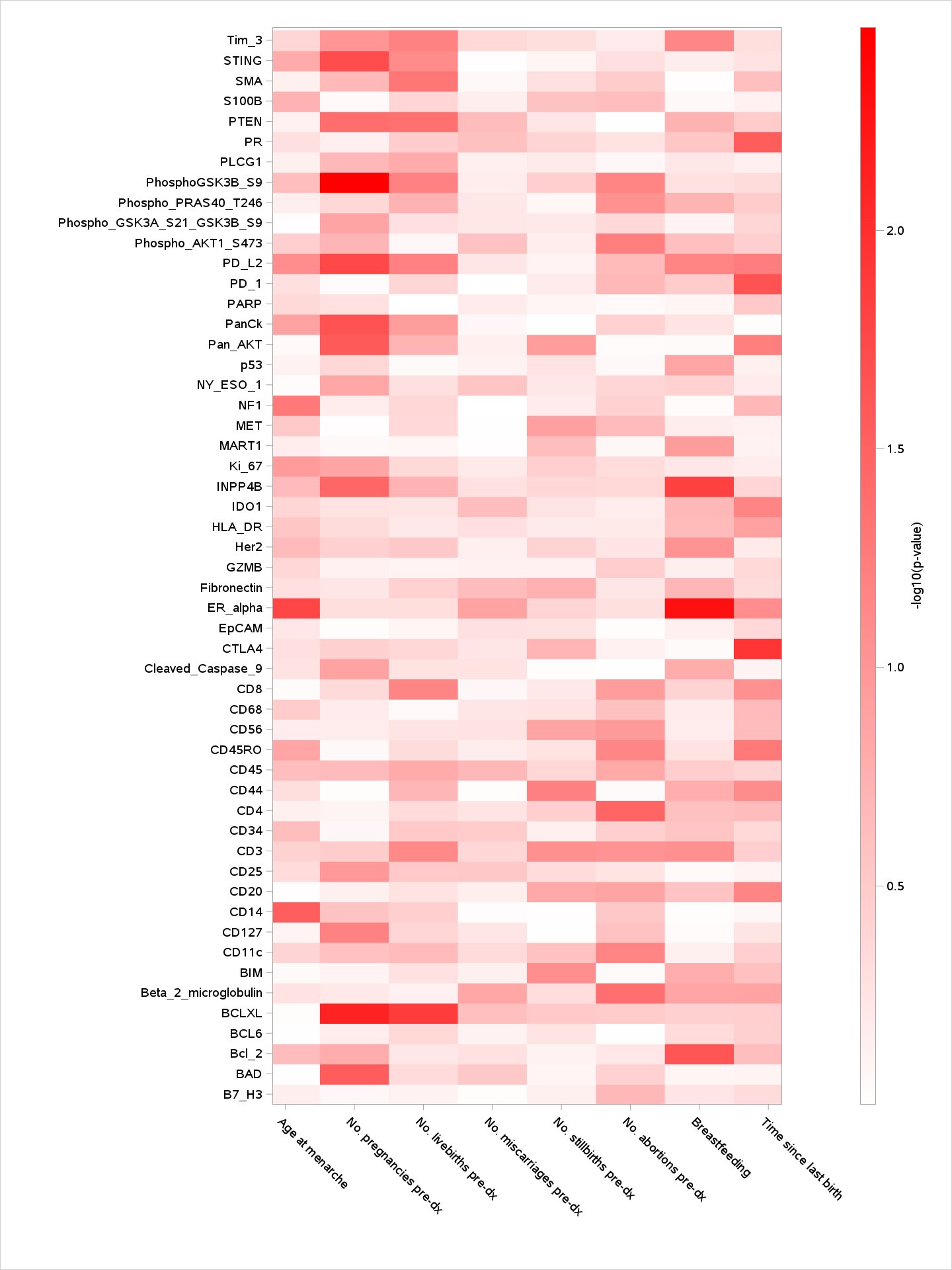
**

**Figure S1 B. Heatmap of reproductive risk factor levels in relation to biomarker levels in keratin-poor segments of ROIs of 336 parous women ages < 40 years**

**
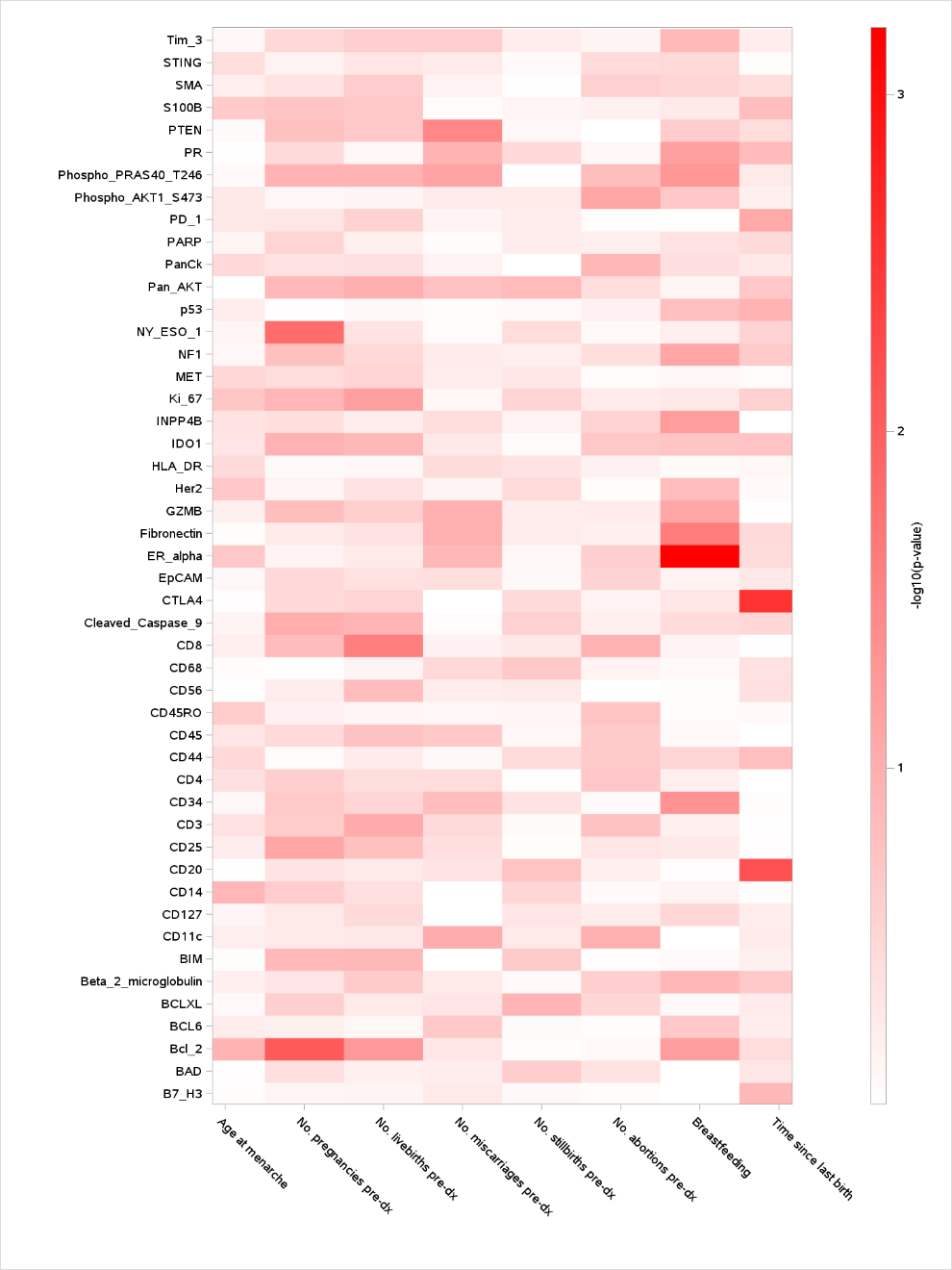
**

**Figure S2**

OPAL analysis of 1,237 TMA cores of breast cancers from women ages 40 years or younger from the Young Women’s Breast Cancer Study. Marker‑positive cells were identified using automated segmentation and phenotype thresholds derived from single‑plex controls. Marker positive cells, standardized by the total number of cells, were natural log- transformed, within CK‑gated epithelial or stromal masks. Associations between TSLB and marker expression were estimated using regression models with parity/TSLB as a predictor (x-axis:1=0-3 years postpartum; 2=3-5 years postpartum; 3=6-10 years postpartum; 4=11-21 years postpartum; 5= nulliparous). Quantification of biomarker expression displayed as boxplots for CK⁺ epithelium (PR, PTEN) and CK⁻/stromal compartments (CTLA4, SMA, CD20) (markers (left to right; y-axis: CTLA4, CD20, SMA, PTEN, PR) by molecular subtype: A: Luminal A; B: Luminal B; C: HER2-enriched and D: Triple-negative.


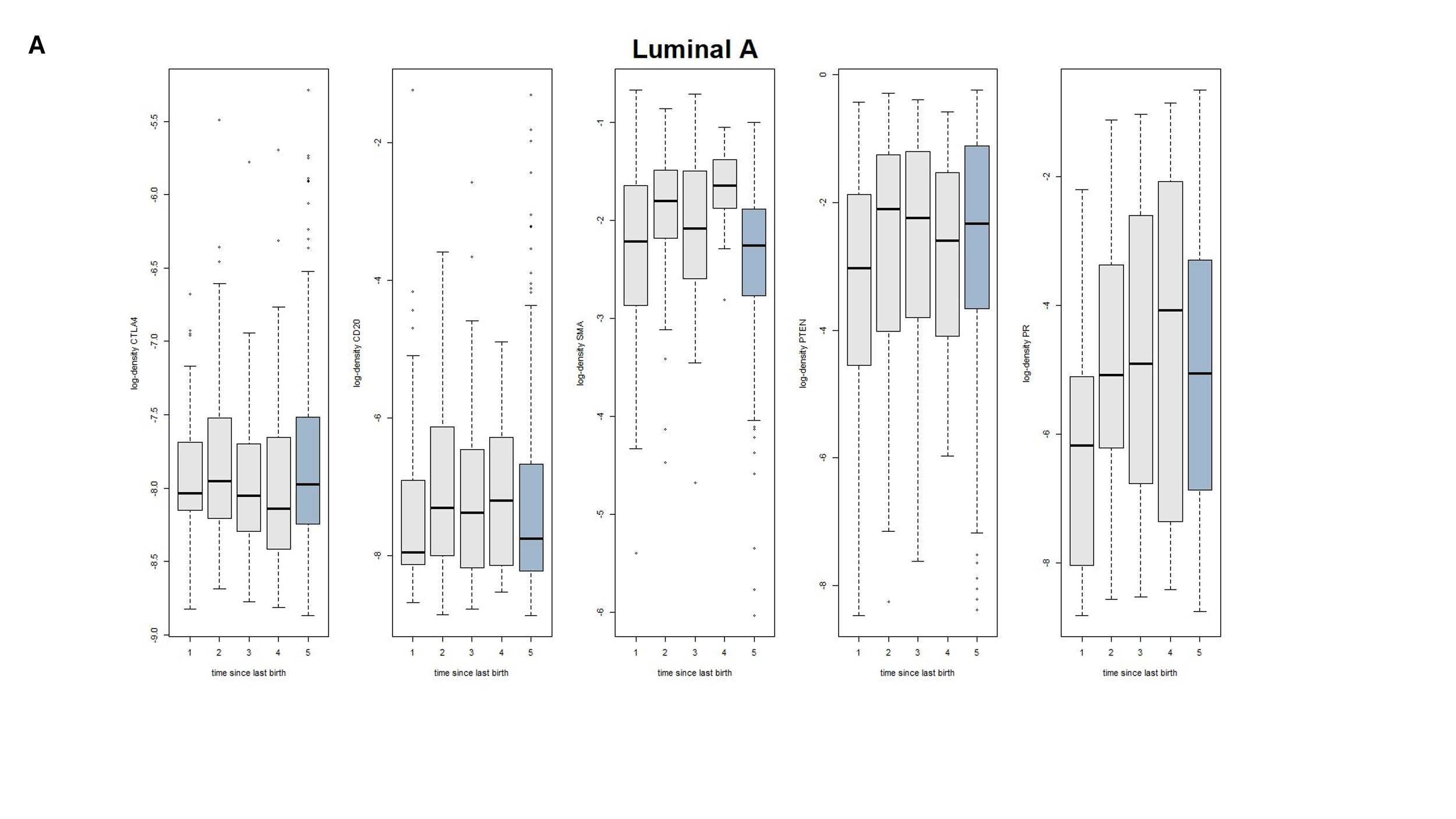


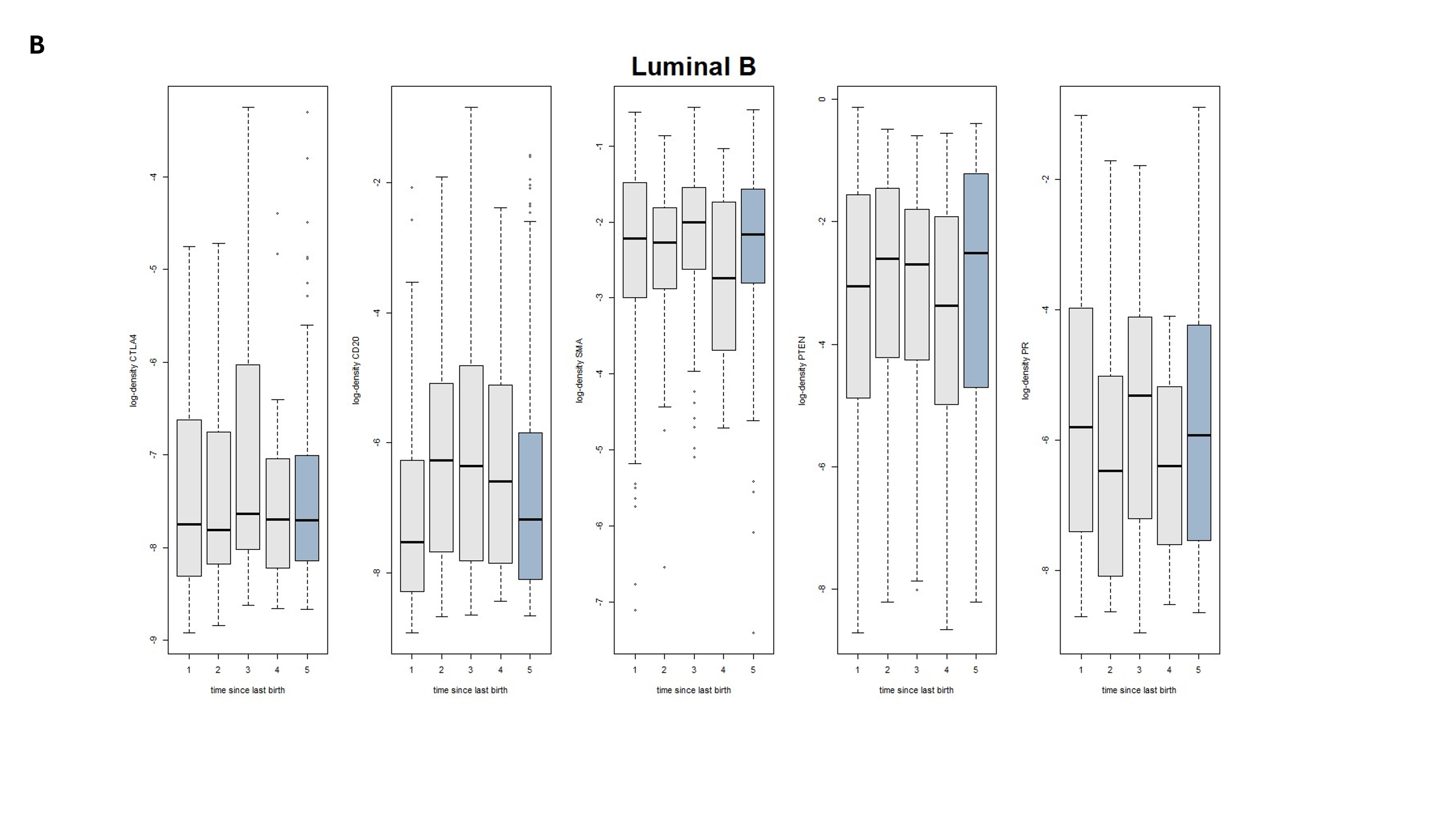


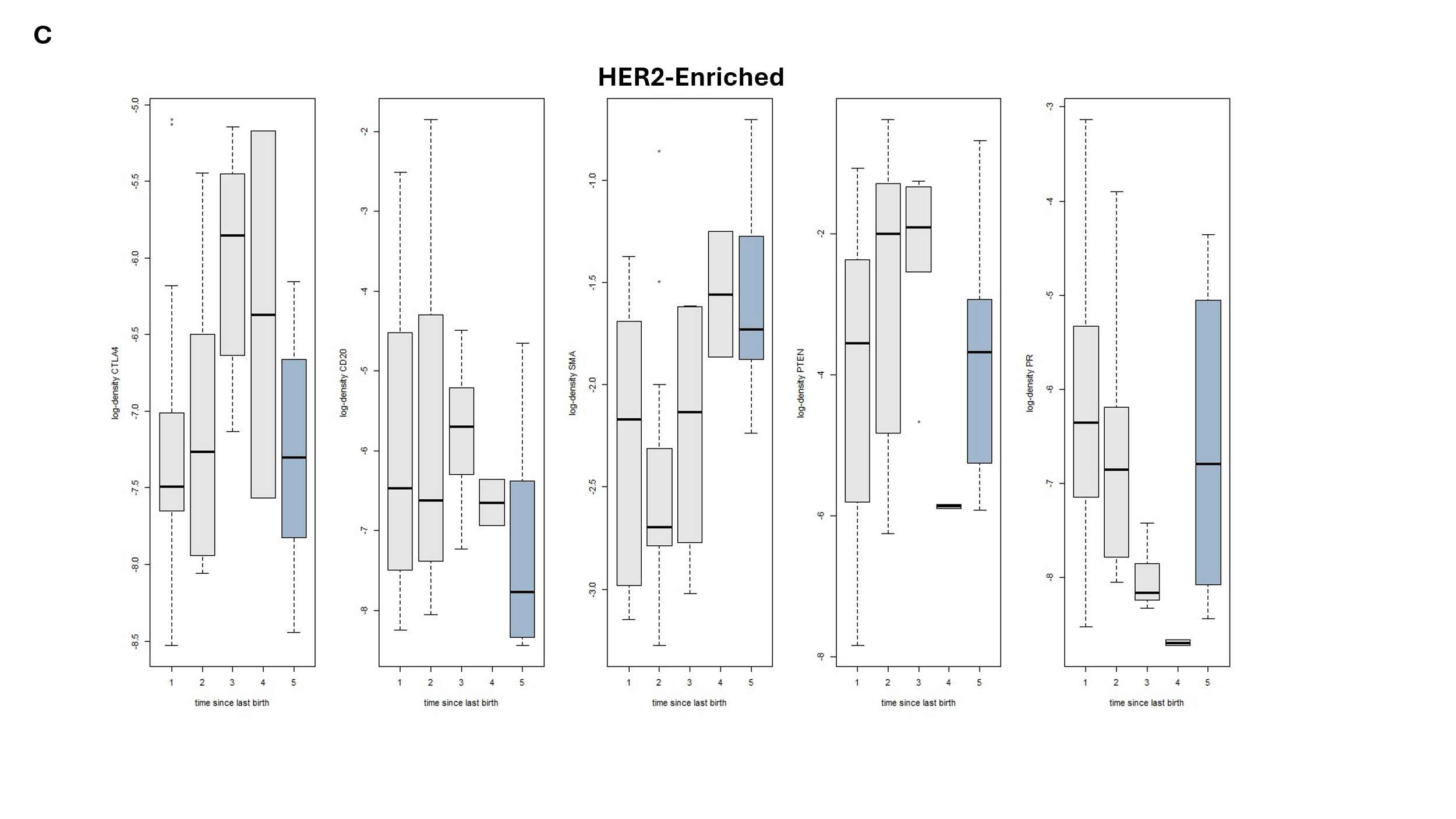


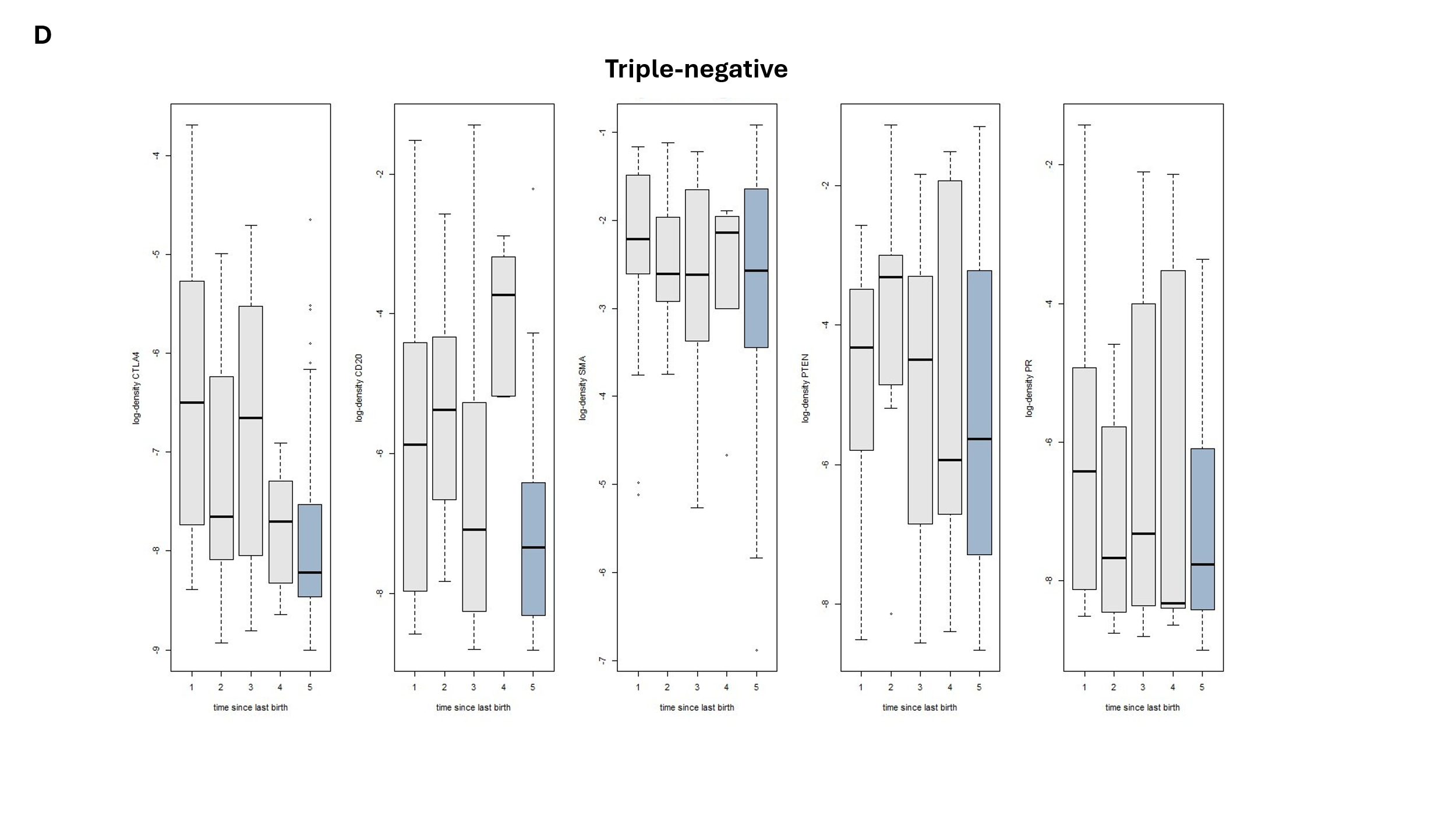


**Figure S3.** Nearest‑neighbor cumulative distribution **G(r)** for CK⁺→SMA⁺CK⁻ interactions in luminal A tumors, 0–50 µm by TSLB category (0–2, 2–5, 5–10, >10, and nulliparous). For a given radius *r*, **G(r)** is the fraction of CK⁺ cells having ≥1 SMA⁺CK⁻ neighbor within distance *r*; higher curves indicate greater epithelial–CAF proximity at a given scale. Curves are shown by TSLB bin; per‑patient aggregation was used prior to bin‑level summaries.


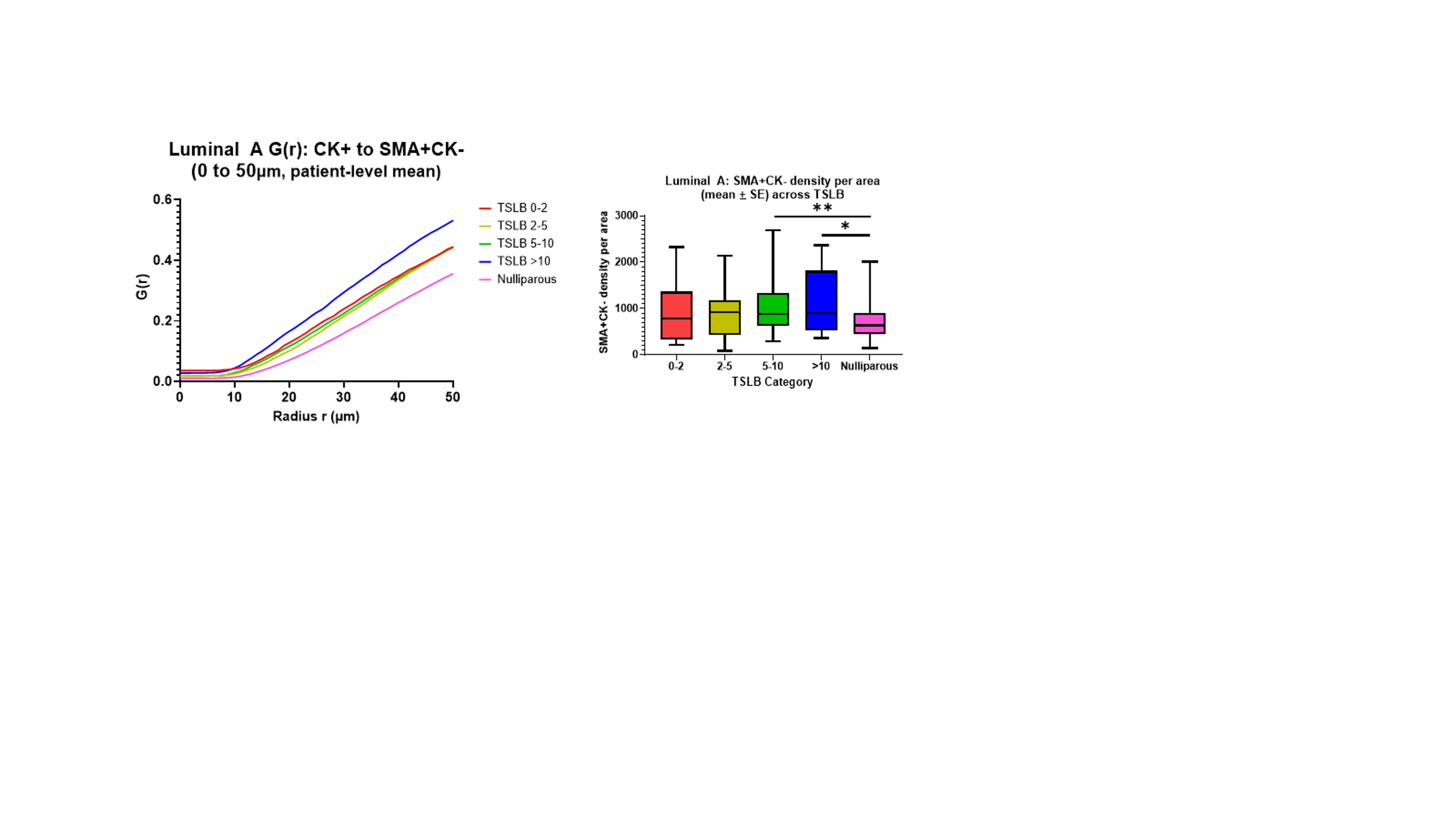


**Figure S4**. Cluster analysis of the 53 biomarker residuals in parous study participants with known time since last birth, in the keratin-rich data set, using Uniform Manifold Approximation and Projection

**Figure S5.** Heatmap of pairwise Pearson correlations of the 53 biomarker residuals in the parous study participants with known time since last birth, in the keratin-rich data set
